# Automated Segmentation and Morphometric Analysis of Thioflavin-S-Stained Amyloid Deposits in Alzheimer’s Disease Brains and Age-Matched Controls Using Weakly Supervised Deep Learning

**DOI:** 10.3390/ijms26157134

**Published:** 2025-07-24

**Authors:** Gábor Barczánfalvi, Tibor Nyári, József Tolnai, László Tiszlavicz, Balázs Gulyás, Karoly Gulya

**Affiliations:** 1 Department of Cell Biology and Molecular Medicine, University of Szeged, 6720 Szeged, Hungary; barczanfalvi.gabor@med.u-szeged.hu; 2 Department of Medical Physics and Informatics, University of Szeged, 6720 Szeged, Hungary; nyari.tibor@med.u-szeged.hu (T.N.); tolnai.jozsef@med.u-szeged.hu (J.T.); 3 Department of Pathology, University of Szeged, 6725 Szeged, Hungary; tiszlavicz.laszlo@med.u-szeged.hu; 4 Department of Clinical Neuroscience, Karolinska Institute, 171 77 Stockholm, Sweden; balazs.gulyas@ki.se; 5 Cognitive Neuroimaging Centre, Nanyang Technological University, Singapore 636921, Singapore

**Keywords:** Alzheimer’s disease, amyloid plaques, class activation mapping, microscopic morphometry, thioflavin-S, weakly supervised semantic segmentation

## Abstract

Alzheimer’s disease (AD) involves the accumulation of amyloid-β (Aβ) plaques, whose quantification plays a central role in understanding disease progression. Automated segmentation of Aβ deposits in histopathological micrographs enables large-scale analyses but is hindered by the high cost of detailed pixel-level annotations. Weakly supervised learning offers a promising alternative by leveraging coarse or indirect labels to reduce the annotation burden. We evaluated a weakly supervised approach to segment and analyze thioflavin-S-positive parenchymal amyloid pathology in AD and age-matched brains. Our pipeline integrates three key components, each designed to operate under weak supervision. First, robust preprocessing (including retrospective multi-image illumination correction and gradient-based background estimation) was applied to enhance image fidelity and support training, as models rely more on image features. Second, class activation maps (CAMs), generated by a compact deep classifier SqueezeNet, were used to identify, and coarsely localize amyloid-rich parenchymal regions from patch-wise image labels, serving as spatial priors for subsequent refinement without requiring dense pixel-level annotations. Third, a patch-based convolutional neural network, U-Net, was trained on synthetic data generated from micrographs based on CAM-derived pseudo-labels via an extensive object-level augmentation strategy, enabling refined whole-image semantic segmentation and generalization across diverse spatial configurations. To ensure robustness and unbiased evaluation, we assessed the segmentation performance of the entire framework using patient-wise group k-fold cross-validation, explicitly modeling generalization across unseen individuals, critical in clinical scenarios. Despite relying on weak labels, the integrated pipeline achieved strong segmentation performance with an average Dice similarity coefficient (≈0.763) and Jaccard index (≈0.639), widely accepted metrics for assessing segmentation quality in medical image analysis. The resulting segmentations were also visually coherent, demonstrating that weakly supervised segmentation is a viable alternative in histopathology, where acquiring dense annotations is prohibitively labor-intensive and time-consuming. Subsequent morphometric analyses on automatically segmented Aβ deposits revealed size-, structural complexity-, and global geometry-related differences across brain regions and cognitive status. These findings confirm that deposit architecture exhibits region-specific patterns and reflects underlying neurodegenerative processes, thereby highlighting the biological relevance and practical applicability of the proposed image-processing pipeline for morphometric analysis.

## 1. Introduction

Alzheimer’s disease (AD) is a progressive neurodegenerative disorder that primarily affects individuals in late middle age or old age and is characterized by a heterogeneous phenotype involving mixed proteinopathies [1,2,3,4]. Although the exact molecular mechanisms of AD remain unclear, its diagnosis relies on characteristic histopathological features. Abnormal alterations observed in AD include extracellular amyloid-β (Aβ) deposits (such as senile, neuritic plaques), neurofibrillary tangles resulting from hyperphosphorylated tau protein aggregation, and cerebral amyloid angiopathy involving the vasculature. Tau pathology in AD progresses predictably across six stages (Braak stages), starting in the transentorhinal and hippocampal regions before spreading to neocortical areas [1,2]. Heterogeneous Aβ plaque forms, derived from amyloid precursor proteins [5,6,7], are also a key AD hallmark and progress in a similar hierarchical pattern (Thal phases), starting in the isocortex and spreading to allocortical, subcortical regions, and eventually to the brainstem [4,8,9,10]. Thus, the reliable detection, segmentation, and quantification of these Aβ plaques in brain tissue are essential for understanding disease progression and evaluating potential interventions. Aβ plaques exhibit considerable morphological diversity, ranging from diffuse, loosely structured deposits with varying size, amorphous shape, and blurred borders, to late-stage, compact fibrillar forms, like mature dense-cored plaques containing central fibrillar Aβ cores with or without a surrounding halo (“core-only” or “burned-out” type). Classification schemes are largely subjective and primarily based on morphological features. Plaques develop progressively, although the precise temporal sequence, influence of regional factors, and their roles in neurodegeneration remain unclear [4,11,12,13,14,15].

Fluorescent amyloid probes, such as thioflavin-S (ThioS), detect Aβ plaques by binding misfolded protein aggregates via β-sheet intercalation (without sequence specificity) both in the parenchymal and vascular amyloid pathology, morphology-based exclusion is commonly used to distinguish these components for qualitative and quantitative assessment [16,17,18,19]. Hereafter, “parenchymal amyloid deposits” refers to plaques and other microscopically detectable pre-amyloid-like structures. ThioS staining is a widely used histological method for labeling Aβ aggregates, yielding intense green-yellow fluorescence (peak 455 nm) under epifluorescence microscopy due to its β-sheet affinity [16]. A previous study has also explored the clinical relevance of the delivery method of aerosolized ThioS probe for in vivo imaging of Aβ deposits in the retina using mouse models, demonstrating its potential for early detection of AD, with the advantage of easy accessibility without the need to cross the blood–brain barrier [20]. Despite its effectiveness, the automated analysis of ThioS-labeled tissue micrographs remains technically challenging, as high-resolution fluorescent imaging often introduces artifacts such as uneven illumination and background fluorescence [21,22], while ThioS protocols using high dye concentrations and short incubation, and solvents may cause non-specific staining (e.g., vessels) and loss from low-affinity sites, underdetecting subtle amyloid [16,17,23]. Furthermore, the heterogeneity in the size, shape, and distribution of amyloid plaques across stages and brain regions make robust segmentation particularly difficult. These issues compromise the reliability and reproducibility of conventional image analysis pipelines, hindering both segmentation and downstream morphometric quantification for neuropathology.

Advances in machine learning and computational pathology, especially convolutional neural networks (CNNs), are addressing limitations in medical image analysis, shifting focus from classification to detection and segmentation for localizing pathological features and delineating anatomical boundaries. CNN architectures like Visual Geometry Group, U-Net, Residual Network, Densely Connected Convolutional Network, Inception, MobileNet, etc., have proven highly effective for these tasks [24]. These models have demonstrated their utility across imaging modalities such as radiological (e.g., X-ray, computed tomography, magnetic resonance imaging), endoscopic, microscopic (e.g., histopathology), ultrasound, ophthalmologic, and other domain-specific medical images, supporting clinical diagnostics [24,25]. Weakly supervised object localization/semantic segmentation (WSOL/WSSS) methods reduce reliance on dense manual annotations by learning from coarse or indirect labels (e.g., holistic image-level class labels), enabling scalable biomedical image analysis with strong segmentation performance. Various studies have demonstrated the applicability of WSOL/WSSS methods to a wide range of tasks, including class activation mapping (CAM)-based techniques [26] for segmenting and analyzing biomedical images [27,28,29,30,31,32,33,34,35,36,37,38,39,40,41]. These approaches improve the ability of models to learn from sparse or weak annotations, contributing to scalable, high-performance biomedical image analysis tools. Moreover, these techniques also play a significant role in enhancing model interpretability. By providing visual insights into which regions of an image influence predictions, CAMs clarify decision-making, which is essential for understanding complex biomedical images [42,43,44,45,46,47,48,49,50,51]. This interpretability is particularly valuable in domains like pathology, where understanding model output reasoning is crucial for clinical application. While similar techniques have been used in AD research and amyloid pathology [52,53,54,55], this study is, to the best of our knowledge, the first to apply a WSSS framework to ThioS-stained amyloid plaques using high-resolution epifluorescence micrographs from human autopsy tissue sections.

To address limitations in current workflows and enable reproducible, automated analysis in AD research, we introduce a scalable, annotation-efficient framework for Aβ-plaque detection and morphometry that integrates established histological methods with modern weakly supervised machine learning strategies.

## 2. Results

### 2.1. Evaluation of the SqueezeNet Classifier for Parenchymal Amyloid Detection

To localize parenchymal amyloid pathology, we first trained a fully supervised binary image classifier based on the SqueezeNet architecture, incorporating a simple bypass structure and a Thresholded Average Pooling (TAP) layer in place of the conventional Global Average Pooling (GAP). The model was trained on patch-level image labels indicating the presence or absence of ThioS-positive parenchymal deposits, enabling downstream CAM extraction for localization tasks. These input patches were derived from histological micrographs of AD patients’ ThioS-stained brain tissue sections. Importantly, the classification model was not used for direct segmentation but served as the basis for extracting CAMs, which were later utilized in the training of separate segmentation models. Accordingly, this sub-section focuses on evaluating the classification performance as a critical preliminary step in the overall WSSS pipeline.

**Figure 1:**
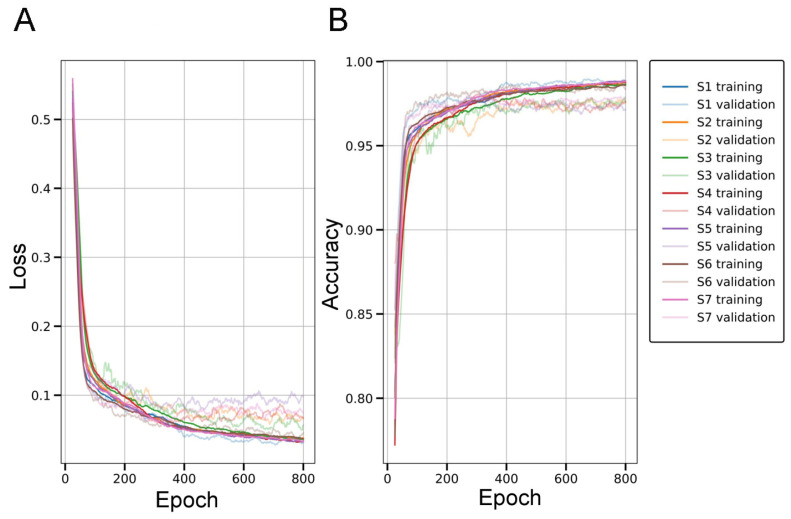
Monitoring performance metrics across patient-wise 7-fold cross-validation of the SqueezeNet model. (**A**) Binary cross-entropy loss ℒ_BC_ and (**B**) accuracy curves are shown for both training and development sets. Each color represents a different cross-validation iteration, where a distinct subject (test fold) was excluded from training. Within each iteration, the remaining data (from the other 6 folds) were split into 90% training and 10% development sets. Darker shades indicate the training sets, while lighter/transparent shades of the same color correspond to their respective developing (validation) sets, used to monitor performance during training. For visualization purposes, the curves are smoothed using a 25-epoch moving average (this helps in observing overall trends, minimizing short-term fluctuations).

**Figure 2:**
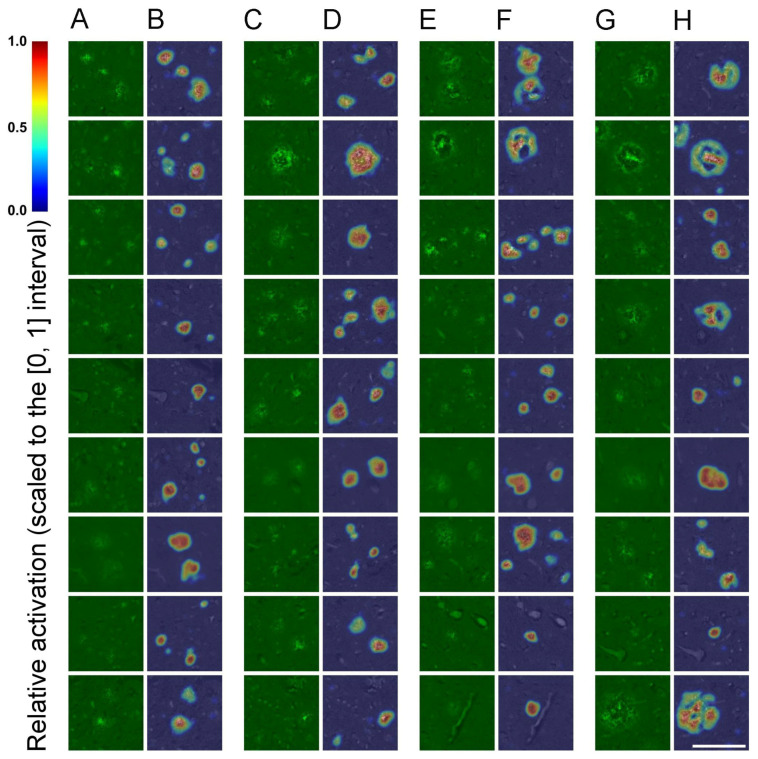
Qualitative evaluation of class activation maps (CAMs) derived from the Thresholded Average Pooling (TAP)-SqueezeNet binary classifier (final model trained on the complete dataset). The figure displays four pairs of alternating columns of (**A**,**C**,**E**,**G**) original input image patches (left) and (**B**,**D**,**F**,**H**) corresponding heatmaps (right), overlaid on the original images. Heatmaps were generated using negative weight-clamping, upsampled to the input image size (572 × 572 pixels), and scaled to the [0, 1] interval. The color bar on the right represents heatmap intensity, ranging from low (blue) to high (red) values, using the “jet” colormap. These maps highlight regions where the model identifies features associated with the presence of parenchymal amyloid structures, which were subsequently used to guide segmentation model training. Scale bar: 100 μm.

To assess generalization across individuals, model performance was evaluated through patient-wise 7-fold cross-validation (i.e., group k-fold), with each fold corresponding to a distinct patient. In each iteration, the model was trained on six folds and tested on the remaining one, ensuring that each subject served as the test case once. This evaluation strategy, designed for practical scenarios, simulates real-world generalization to unseen individuals while avoiding data leakage and subject-specific overfitting. Within each training run, 10% of the training data (randomly sampled from the six training folds) was held out as a development set to monitor performance and mitigate overfitting (Figure 1). The model achieving the lowest development (validation) loss across a maximum of 800 training epochs was retained and subsequently evaluated on the corresponding test fold. Evaluation metrics were computed for each fold, and the average results across all folds are summarized in Supplementary Table S1. The SqueezeNet classifier achieved a mean accuracy of 97.94%, precision of 98.53%, recall of 96.99%, and F1-score of 97.74%, demonstrating high reliability in distinguishing image patches with or without parenchymal amyloid structures. Supported by consistent and precise predictions, the classifier reliably captured image-level features of parenchymal amyloid pathology under full supervision, enabling CAM-based localization cues for the subsequent segmentation stage, as confirmed by classification results and visual inspection (Figure 2).

### 2.2. Evaluation of the U-Net for Parenchymal Amyloid Segmentation

To enable pixel-level segmentation of parenchymal Aβ deposits in full-image, ThioS-stained micrographs, the U-Net model was trained using CAM-derived binary pseudo-labels generated by a previously trained SqueezeNet distinguishing foreground (FG; amyloid structures) from background (BG; all other tissue or image regions). Although the training was conducted under weak supervision, the final evaluation was performed using manually annotated ground-truth (GT) masks that were never used during training.

For each fold in the 7-fold patient-wise cross-validation, CAMs were extracted from the corresponding SqueezeNet models; they were then postprocessed into pseudo-masks via two-level thresholding, followed by extensive object-level augmentation: FG objects were inpainted out and randomly reinserted, modifying boundaries and spatial context to expand the training distribution. Notably, the development set for each fold also originated from CAM-based pseudo-masks and underwent identical on-the-fly augmentations, ensuring that performance metrics reflected predictions on fully processed inputs, rather than raw (i.e., thresholded) CAMs. Only the test set, which contained the full images, relied on true manually annotated GT masks, kept strictly isolated from the training data, and processed in a patch-based manner.

**Figure 3:**
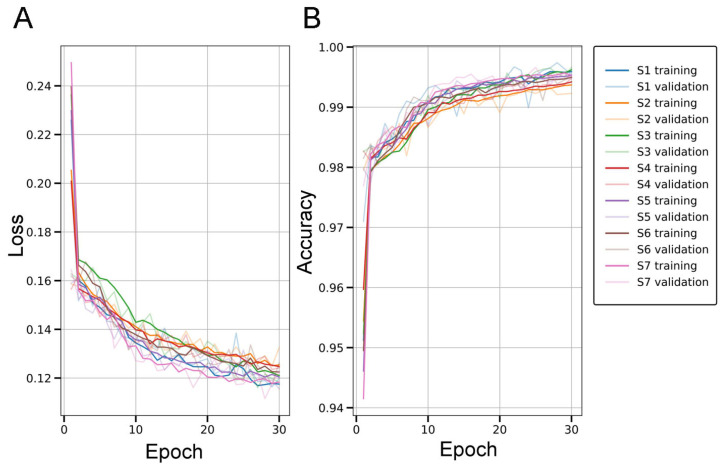
Monitoring of performance metrics of the U-Net segmentation model across patient-wise 7-fold cross-validation. (**A**) Asymmetric unified focal loss ℒ_aUF_ and (**B**) pixel-wise accuracy (PA) curves are shown for both training and development sets. Each color represents a different cross-validation iteration, where a distinct subject (test fold) was excluded from training. Within each iteration, the remaining data (from the other 6 folds) were split into 90% training and 10% development sets. Darker shades indicate the training sets, while lighter/transparent shades of the same color correspond to their respective developing (validation) sets, used to monitor performance during training.

The U-Net models were trained fold-wise, maintaining strict patient-level separation between training and testing data. For each of the seven folds, the model was trained on six folds and tested on the remaining one. During training, 10% of the data was allocated to a development set solely for tracking stable loss and accuracy trends (Figure 3), while model snapshots were saved exclusively at the end of the 30th epoch, regardless of the development set performance.

Across the seven folds, the segmentation model achieved an average Dice similarity coefficient of 0.763, Jaccard index of 0.639, recall of 0.721, and precision of 0.877. Pixel-wise accuracy (PA) and specificity were high at 0.990 and 0.997, respectively; however, due to the strong FG-BG imbalance inherent in amyloid segmentation, these metrics are less informative. The model also yielded a false positive rate of 0.003, a false discovery rate of 0.123, and a false negative rate of 0.279 (Supplementary Table S2).

**Figure 4:**
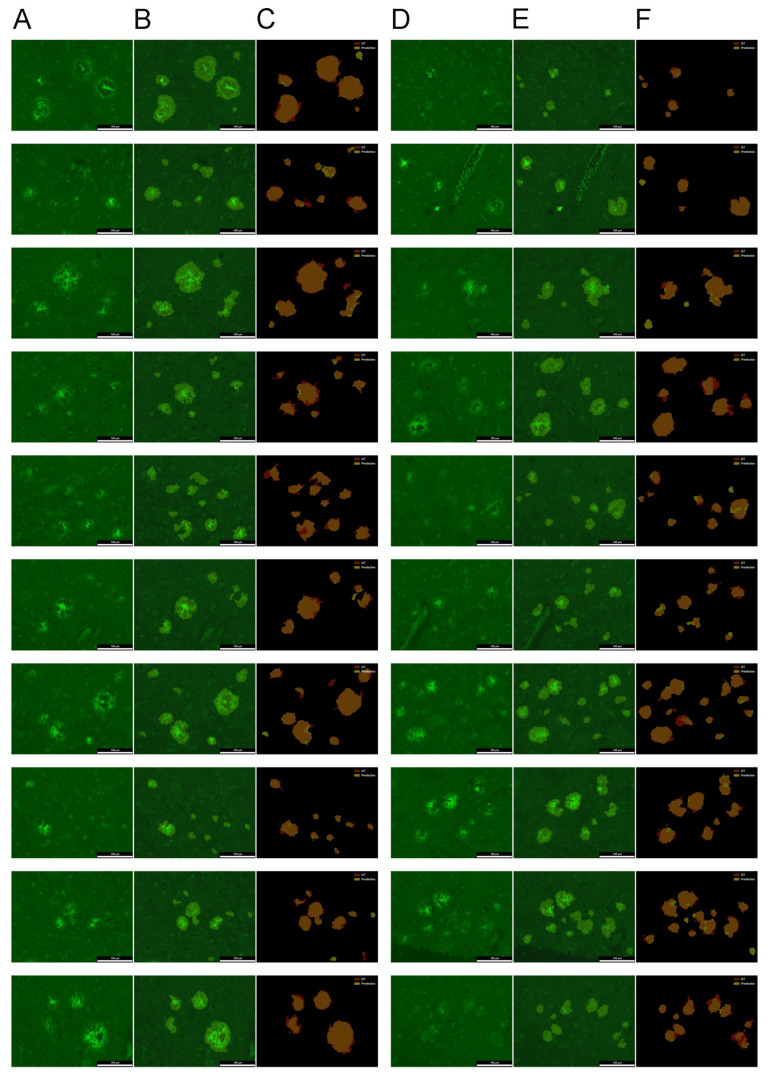
Qualitative evaluation of U-Net binary foreground–background (FG-BG) segmentation results (final model trained on the complete dataset). Three sets of columns are shown: full-size input images (**A**,**D**), corresponding postprocessed predictions (generated in a patch-wise manner) overlaid on the input images (**B**,**E**), and segmentation outputs overlaid on ground truth (GT) masks using transparent blending (**C**,**F**). The FG pixels/segment in the GT are marked in red, predicted FG in yellow, and the overlap appears in orange, indicating agreement. Small isolated red and yellow regions can be seen upon closer inspection, mostly near vessels, clearance zones, and objects we could not identify. The model accurately captures diverse plaque morphologies and structural features, while effectively distinguishing these from vascular regions. Despite their high fluorescence intensity, vascular regions are correctly classified as BG, effectively separating parenchymal pathology from the vasculature and BG tissue features based on their semantic differences, which the ThioS-staining method alone cannot distinguish. Scale bar: 100 μm.

Taken together, our results show that dense segmentation of parenchymal amyloid structures can be effectively approximated under weak supervision and object-level augmentation, without manual pixel-level annotations. Despite relying solely on CAM-derived pseudo-labels, the model maintained robust performance across folds against human-annotated GT (Figure 4). This demonstrates a viable and scalable strategy for generating reliable histopathology maps from image-level labels using CAMs, object-level image synthesis, and robust data augmentation, with minimal manual annotation effort.

### 2.3. Morphometric Profiling of Amyloid Plaques via Particle Analysis

To assess morphological characteristics of ThioS-stained amyloid deposits across brain regions and cognitive statuses, we used a final segmentation model trained on the entire available dataset to generate binary masks for detailed particle analysis. Unique deposits were extracted as connected components, followed by morphometric measurements (Supplementary Data) with ImageJ (version 1.47) and the FracLac plugin (for details, see https://imagej.net/ij/plugins/fraclac/FLHelp/Introduction.htm; accessed on 15 February 2025).

**Figure 5:**
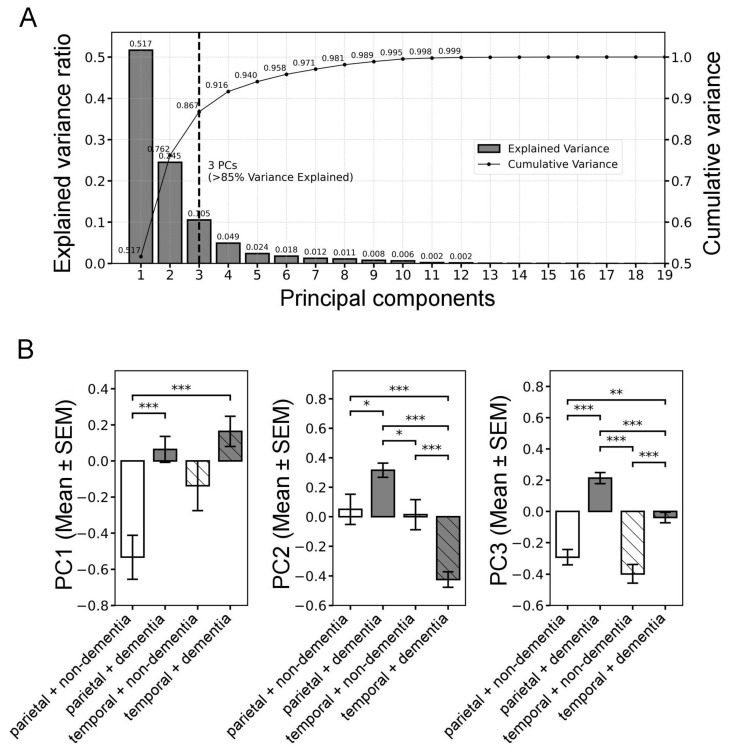
Principal component analysis (PCA) and group-wise comparisons reveal major axes of morphological variation in ThioS-stained plaques and highlight region- and dementia-specific differences. (**A**) Scree plot showing the explained variance (bars) and cumulative variance (line) by PCA principal components across the 19 extracted morphometric features. The first three principal components (PCs) account for over 85% of the total variance, with PC1 alone explaining more than 50%. (**B**) Group-wise comparisons of the first three PCs, representing size-related (PC1), structural complexity (PC2), and geometric symmetry properties (PC3). Bar plots show mean ± SEM of PC scores per plaque, grouped by region (parietal = solid, temporal = striped) and cognitive status (dementia = dark, non-dementia = light). Grouping is based on the donor subject. Significant differences across groups are denoted by asterisks (* *p* < 0.05, ** *p* < 0.01, *** *p* < 0.001), revealing consistently higher PC scores in the parietal + dementia group across all components. These results suggest that plaques in individuals with dementia, particularly in the parietal cortex, are larger, more structurally complex, and geometrically more irregular, although regional differences are also apparent.

The measured parameters were then analyzed across groups based on the donor’s brain region and dementia status. Principal Component Analysis (PCA) was conducted on 19 morphological parameters of the deposits, revealing three principal components (PCs) that together explained over 85% of the total variance in the dataset (Figure 5A). Component loadings reflect standardized coefficients of the original variables, indicating their relative contribution to each PC (Supplementary Table S3).

The first component (PC1) primarily loaded on overall size-related descriptors, including deposit area (0.315), perimeter (0.287), compact area (0.274), hull area (0.304), and mean radius (0.291). This component captures the overall deposit size and extent. Integrated density (0.314), while intensity-based, is closely tied to area (total signal), thus aligning with size-related parameters. PC2 was mainly associated with shape complexity metrics, dominated by solidity (0.395), circularity (0.372), fractal dimension (0.313), and lacunarity (−0.370). Diffuseness index (−0.310), though intensity-based, reflects the spatial spread of intensity and contributes to morphological complexity. PC3 was driven by global geometric features, particularly span ratio (0.520), max/min radii (0.469), and hull circularity (−0.511), characterizing overall elongation and spatial arrangement. Group-wise comparisons of component scores revealed significant regional and cognitive-status-dependent differences. One-way analysis of variance (ANOVA) followed by Tukey’s post hoc test indicated that all three components differed significantly between the groups (PC1: F = 7.29, *p* < 0.001; PC2: F = 33.72, *p* < 0.001; PC3: F = 35.82, *p* < 0.001) (Figure 5B, Supplementary Tables S4 and S5). PC1 scores were significantly higher in the parietal + dementia and temporal + dementia groups compared to parietal + non-dementia (*p* < 0.001), suggesting an overall increase in deposit size in the parietal cortex of individuals with dementia. PC2 scores were elevated in parietal + dementia relative to all other groups (all *p* < 0.05), reflecting increased texture irregularity and complexity. Interestingly, the strongest contrast was observed between parietal + dementia and temporal + dementia (*p* < 0.001), while PC2 scores were significantly lower in temporal + dementia compared to all other groups (*p* < 0.001), suggesting region-specific morphological divergence in the presence of dementia. PC3 scores displayed robust group differences, with parietal + dementia consistently scoring higher than all other groups (all *p* < 0.001), pointing toward expanded radial structure and elongation of deposits. The temporal + dementia group also showed elevated scores compared to both groups without dementia (all *p* < 0.01), although scores remained lower than those observed in the parietal + dementia group (*p* < 0.001). Within each brain region, groups with dementia showed significantly higher scores than the corresponding groups without dementia (all *p* < 0.001), reinforcing the relevance of PC3 in capturing dementia-associated morphological traits.

Altogether, these findings suggest that distinct components of deposit morphology (PC1, PC2, and PC3) differ significantly across both brain region and cognitive status, supporting the hypothesis that deposit architecture is not only regionally specific but also reflects underlying neurodegenerative processes. However, due to the limited number of donor cases, these PCA and group-wise comparison results should be interpreted with caution and are primarily intended to serve a demonstrative and exploratory purpose.

## 3. Discussion

### 3.1. SqueezeNet-Based CAM for Weakly Supervised Localization

In this study, SqueezeNet was used as the backbone for a fully supervised image-level classification task and as the base of the WSOL framework, enabling the identification of parenchymal amyloid deposits from histopathological micrographs. To improve coarse localization, the architecture was complemented with a TAP layer, allowing effective extraction of CAMs using image-level labels alone.

While CNNs are typically optimized to maximize accuracy, smaller models that retain strong performance offer practical benefits, particularly in resource-constrained environments. SqueezeNet, a compact, modular CNN architecture for classification tasks, known for achieving performance comparable to AlexNet with a ~50× reduction in parameter count [56], has been used to extract CAMs. To reduce complexity and parameter count, SqueezeNet employs ‘fire modules’ that favor 1 × 1 filters over 3 × 3 filters and reduce the size of layers containing 3 × 3 filters by limiting their input channels. Delayed downsampling preserves spatial detail by maintaining larger activation maps in deeper layers, while the global pooling layer further aids regularization, enhances spatial robustness, and strengthens the connection between feature maps and categories [56], making SqueezeNet inherently suitable for CAM.

In standard CNN classifiers, convolutional layers act as feature extractors, and the final feature representations, obtained by vectorizing the last convolutional layer feature maps, are typically classified using fully connected layers with softmax or sigmoid activation [57]. As an alternative, global pooling layers convert activation maps into fixed-size vectors by aggregating spatial information across feature maps. This reduces overfitting and enhances generalization while preserving the convolutional structure and improving interpretability by linking feature maps to categories [57]. Despite potential trade-offs in accuracy and task-limited applicability [58], global pooling is especially valuable in domains like medical imaging, where transparent and explainable predictions are essential. The most common global pooling operations are GAP and Global Max Pooling (GMP). GAP is often preferred for localization tasks as it averages the entire feature map, maximizing the output score when the object is fully activated, making it effective for capturing the full extent of an object [26]. However, it may suppress highly activated regions, potentially affecting classification performance [59]. In contrast, GMP focuses on the strongest activation, making it better suited for identifying the most discriminative regions, as lower-activation areas do not influence the result. Studies related to the CAM technique have explored similar approaches using GMP [60] and log-sum-exp pooling [61]. Additional global pooling operations have been developed to address emerging challenges, such as Global Average of Top-K Max-Pooling [62], Top-GAP [63], Deep Generalized Max-Pooling [64], and TAP [59].

Generalized CAM methods, like Grad-CAM [58], Grad-CAM++ [65], Score-CAM [66], Poly-CAM [67], and HR-CAM [68], explore the decision-making of an arbitrary CNN. Grad-CAM uses output gradients to weight the feature maps, offering broad applicability across computer vision tasks without requiring specialized architectures [58], while Grad-CAM++, using scaling factors derived from higher-order gradients, improves localization when multiple class instances are present [65]. Still, gradient-based methods are computationally intensive, while mapping resolution also depends on the last convolutional layer, and resolution-enhancing solutions may reduce transparency, especially when involving multiple layers, making global pooling a more common choice in WSOL [59]. We opted for a simpler CAM method with a TAP layer, as the primary goal was to train a binary image classifier to detect parenchymal amyloid structures, rather than solving more complex computer vision tasks. Notably, SqueezeNet, being fully convolutional, requires no major architectural changes (e.g., removing fully connected, convolutional, or pooling layers) to apply CAM and achieve appropriate mapping resolution, making it particularly well-suited for this purpose.

Although the focus here is on evaluating classification performance, architectural and pooling choices were driven by the downstream need for spatial localization. Conventional CAM approaches, such as those based on GAP, are known for their ease of implementation but often suffer from insufficient activation spread [58]. Replacing GAP with TAP allowed us to retain high activation specificity without discarding lower-activation regions that could still carry clinically meaningful information. This was particularly relevant given the subtle and variable nature of ThioS staining, where pathology could also manifest in diffuse or weakly marked regions.

Notably, direct quantitative evaluation of the CAM-derived pseudo-labels (e.g., via ablation study) was not performed, as both patch-level assessment and full-image reconstruction would require substantial additional complexity (e.g., postprocessing or architectural modifications) and offer only limited interpretability due to conceptual and technical constraints, stemming from the binary-labeled, patch-based dataset structure, partial tile overlap, relative scaling of CAM activations, two-level thresholding and resolution mismatch, all of which introduce FG-BG distribution bias, redundancy, and challenges in intensity alignment (i.e., seamless integration), particularly in handling negative (plaque-free) regions.

Our results confirmed that SqueezeNet with skip connections (Simple Bypass) and a TAP layer learned discriminative representations from a relatively small and heterogeneous dataset (Figure 2), demonstrating robust classification in subject-wise 7-fold cross-validation, suggesting good generalization to unseen individuals (Supplementary Table S1). This is a critical requirement in medical applications, where variability across patients often exceeds that within samples from a single subject. Avoiding gradient-based CAM methods (e.g., Grad-CAM, Score-CAM) was motivated by the need for computational simplicity and architectural compatibility. While these methods can improve localization, they require backpropagation gradients during inference, limiting real-time or large-scale use. In contrast, the TAP-based solution yielded heatmaps without incurring these computational costs or architectural dependencies. However, as with all weakly supervised methods, one major limitation is the trade-off between localization accuracy and classification performance. Despite optimizing the pooling strategy, the final CAMs remained limited in spatial resolution due to the downsampling inherent in deep convolutional layers. While we partially mitigated this by increasing the input resolution (572 × 572) and leveraging SqueezeNet’s late downsampling strategy, the resulting activation maps still lacked precise boundary definitions, a known bottleneck of CAM-based localization. Therefore, these CAMs were used as coarse initializations for the subsequent segmentation stage, rather than as final segmentation outputs.

Our model achieved strong classification performance metrics, including a mean accuracy of 97.94%, precision of 98.53%, recall of 96.99%, and an F1-score of 97.74%, highlighting its effectiveness in distinguishing image patches with versus without parenchymal amyloid pathology. Notably, these results were validated through patient-wise cross-validation, which confirmed the model’s robustness and generalizability across patients. This cross-validation approach is particularly valuable in ensuring that the model performs consistently and accurately, even when exposed to previously unseen patients. The findings suggest that the classifier is not only reliable in controlled experimental settings but also applicable to real-world histopathological scenarios, where variability between patients is inevitable.

In summary, the SqueezeNet + TAP framework provided a practical and efficient solution to generate interpretable activation maps suitable for guiding downstream weakly supervised segmentation stages and refinement with minimal annotation needs, while simultaneously achieving competitive classification performance. This phase established the groundwork for refinement and object-level morphometric analysis in the subsequent stages of our pipeline.

### 3.2. U-Net Segmentation with Advanced Object-Level Augmentation

Our findings demonstrate that weak supervision, when combined with targeted object-level augmentation, can effectively train deep segmentation models to delineate parenchymal amyloid deposits. Despite relying on training labels derived from weakly annotated images, the U-Net model generalized well to manually annotated GT across folds. Our results highlight the capacity of synthetic training data, generated via modular object–BG recombination, to capture real biological structures and refined boundaries with high fidelity.

WSSS with image-level labels aims to generate dense pseudo-masks by classification models, typically via CAMs, that highlight semantically relevant image regions. These initial regions are then refined using postprocessing techniques and/or transformed into pseudo-masks to train segmentation networks, though their inherent coarseness poses challenges for learning under noisy/imperfect supervision [69,70]. Segmenter CNNs often struggle with precision due to architectural constraints like spatial invariance and smoothing effects, exacerbated by class imbalance, uncertainty, and overfitting on label noise, resulting in oversmoothed or inaccurate class transitions. Boundary recovery, applied after classification, localization, and segmentation, refines coarse predictions by sharpening edge pixels (i.e., class transitions), improving accuracy without altering semantics, typically through postprocessing that enforces local coherence and aligns predictions with anatomical contours [70]. Superpixel-based methods partition images into perceptually coherent regions to refine CNN segmentations by sharpening edges, either as postprocessing or auxiliary training cues; however, their effectiveness depends on manually selected granularity, limiting scalability [70,71]. Conditional Random Fields also serve as postprocessing modules [33] or integrated differentiable layers [72], improving localization and spatial smoothness but requiring separate optimization or adding computational overhead with increased model complexity. Multiscale architectures fuse shallow and deep features to enhance boundary detail [32], and boundary-aware loss functions guide training to emphasize pixel-level accuracy at class transitions [73,74,75]. Rather than relying on a single noisy proposal, some WSSS methods exploit multiple annotation candidates as joint multilabel supervision, enabling the model to capture shared semantics and reduce overfitting [76]. Soft labeling, originally designed for classification, has been adapted to image segmentation to handle ambiguity, inter-class confusion, observer variability, poorly defined regions and prevent overfitting on noisy labels. Beyond simple uniform or Gaussian-based label smoothing, segmentation methods apply soft labels in a spatially structured manner (e.g., dilated masks, spatially varying smoothing, or geodesic distance-based weighting) to better reflect spatial structure and image context [77,78,79,80]. Our approach likewise focuses on refining coarse pseudo-masks by training a U-Net with object-level augmentation and contour-perturbation-based image synthesis.

U-Net, a widely used fully CNN architecture introduced by Ronneberger et al. [75], was originally designed for biomedical image segmentation and is now applied across various domains. It excels in several modalities, including electron microscopy, magnetic resonance imaging, and computer tomography scans, for identifying tissues, tumors, and anatomical structures, and has also been successfully applied to general image segmentation tasks beyond medical applications [81,82,83]. Designed to handle limited annotated data common in medical imaging, it uses a symmetric encoder–decoder architecture to enable precise segmentation. The encoder captures contextual information through repeated unpadded 3 × 3 convolutions and 2 × 2 max pooling, gradually downscaling spatial dimensions and expanding feature depth. The decoder reverses the encoding steps by applying transposed convolutions for upsampling and concatenates cropped feature maps from the encoder via skip connections to recover fine spatial details. Cropping is required due to the unpadded convolutions, which reduce feature map size, ensuring that encoder and decoder features align correctly. The network concludes with a 1 × 1 convolution to produce pixel-wise class probabilities, generating accurate segmentation masks while avoiding border artifacts [75,81].

Despite the exceptional performance of modern deep learning models in computer vision, data augmentation remains essential to improve robustness, generalization, and data efficiency. Acting as a powerful regularization, augmentation helps mitigate overfitting, address domain shift (distribution inconsistencies), and alleviate issues like data scarcity, class imbalance, and limited representativity by generating high-quality synthetic/virtual training examples, enhancing dataset volume, diversity, and realism, making models more resilient to real-world variability [84,85]. Beyond classification, it plays an essential role in detection, localization, and segmentation, particularly in high-stakes, annotation-heavy domains like medical imaging and microscopic analysis, where manual labeling is costly, time-consuming, and expertise-bound. Far from being auxiliary, augmentation is now a core component of modern deep learning pipelines [85,86,87]. Within this context, transformation-based input space augmentations can be broadly divided into classical and advanced strategies. Classical methods include geometric transforms (e.g., rotation, flipping, translation, shearing, scaling, cropping, affine/projective warping, non-linear deformations) and non-geometric manipulations (e.g., noise injection, filtering and photometric/color space adjustments like brightness/contrast changes or jittering) [85,86]. Advanced strategies include region- and pixel-level mixing or erasure, alongside data-driven, meta-learning, and automated techniques that mark the frontier of current augmentation research [84,85,86,87]. Image-mixing augmentation blends one or more samples and their labels either pixel-wise (e.g., weighted averaging) or patch-wise [88]. A foundational method, MixUp [89], interpolates between two images and labels using a mixing ratio from a beta distribution, inspiring variants like Manifold Mixup [90] and patch-based approaches such as CutMix [91]. Later refinements improve patch boundaries, exploit saliency or activation maps, and mix one or multiple images via novel strategies, enhancing generalization and robustness against noise or adversarial data across tasks like classification, localization, segmentation, and weakly supervised methods (WSOL, WSSS) [88].

To tackle challenges in weakly supervised learning and semantic segmentation, advanced augmentations manipulate object-level and contextual features to generate more diverse and informative samples, reduce overfitting, and improve robustness. ClassMix [92] blends unlabeled images using masks from network predictions, considering object boundaries, Context Decoupling [93] separates objects from BGs to emphasize object-specific cues, while LCAMix and HSMix [94,95] employ superpixels (homogeneous regions) to maintain local structure and contour integrity in medical image mixing, countering isotropy. Object-aware methods like Cut-Paste-and-Learn (instance detection) [96] extract and store (data banks) objects and BG scenes, apply geometric transforms, and blend inserted objects using Poisson or Gaussian smoothing to reduce boundary artifacts, while the method called Simple Copy-Paste [97] simplifies this by randomly pasting objects between images without modeling visual context, addressing data needs and aiding long-tail category balancing, with semi-supervised learning integration possible. ObjectAug [98] addresses object–BG connectivity, class imbalance, and boundary augmentation by applying classical augmentations to separated objects using semantic labels, and image inpainting for gap-filling, KeepMask and KeepMix [99] vary BGs while preserving FGs to avoid overfitting in medical segmentation and TumorCP [100] uses randomized Copy-Paste with Gaussian blur, demonstrating the effectiveness of inter-patient augmentation. Soft-Copy and Soft-Paste (Soft-CP) [101] improve medical segmentation by using a new object-blending method with erosion/dilation to smooth object edges and adjust relevance based on distance, unlike Poisson or Gaussian smoothing, preserving lesion structure without distorting medical information, while also applying object- and image-level transforms to lesions and BGs to enhance diversity.

ROI contour modification has previously been explored in radiomics, not for segmentation itself, but to improve the robustness of quantitative imaging feature extraction under delineation uncertainty [102,103], in response to two critical challenges: the limited size and class imbalance of datasets, and the observer variability in manual ROI delineations that undermines feature reliability. To address these issues, recent studies have introduced geometric perturbation-based augmentation pipelines operating directly on ROI masks, offering an alternative to conventional feature-level resampling, applying stochastic geometric transformations such as erosion, dilation (volume adaptation), translation, and particularly contour randomization to simulate interobserver variability and evaluate feature stability and discriminative power. By introducing clinically plausible boundary variations without the need for multiple expert annotations, these ROI perturbations mimic natural delineation inconsistencies and uncertainty, augment sample size and diversity, enhance feature robustness, improve the generalizability of radiomic classification models, and aid the reliable recognition of clinically relevant patterns [102,103].

Inspired by these prior works with object-level strategies, we adapt the concept of synthetic contour generation commonly used in texture segmentation tasks, including applications in medical imaging such as hematoxylin/eosin lymphoma mosaics [104], at the object-level. Specifically, we randomize the keypoints of simple, rounded borders extracted from high-confidence CAM regions, while preserving their internal core structure. These transformed objects are then embedded into novel BG scenes using Soft-CP, which mimics natural-looking blending, while uncertain regions are inpainted to suppress noise. This process generates synthetic yet anatomically plausible FG-BG transitions and shape variations. Our method mitigates the model’s tendency to learn overly smooth boundaries caused by upscaled CAM artifacts by exposing it to diverse contour alterations during training, thereby promoting better generalization to unseen real spatial configurations at inference. Separation of images into discrete FG and BG components enabled combinatorial augmentation, vastly expanding the diversity and enhancing the network capacity to learn both fine-grained contours and broader contextual cues. This is reflected in the model’s impressive precision (0.877) and recall (0.721), suggesting a strong ability to accurately differentiate between deposit and non-deposit regions.

Despite the use of ℒ_aUF_ and object-level augmentations favoring the rare FG class, Recall (0.721) and FNR (0.279) values indicate that some deposits remain undetected, like extremely faint or fragmented structures, but this is not unexpected, as the model was not exposed to manually annotated data. Another factor contributing to the mild under-segmentation is the consistent separation of deposits from adjacent vascular elements since the ImageJ-automation and manual editing-based GT masks were less refined in this regard. Post hoc visual inspection confirmed that many of the unsegmented areas corresponded to embedded or directly contacting blood vessels or ambiguous structures that are challenging to delineate, despite efforts to exclude them during annotation, suggesting missing regions were not due to model bias but rather reflect challenges in the GT itself, like imprecise vessel–deposit boundaries. Furthermore, the strict CAM thresholds may have contributed to the exclusion of some ambiguous or borderline features. The model’s exceptionally low false positive rate (FPR = 0.003) confirms its ability to exclude BG and vasculature, enabling parenchyma-specific amyloid detection. This focused identification supports accurate isolation of the parenchymal Aβ fraction, facilitating in-depth analysis of plaque pathology and supporting research into targeted therapies.

The model’s performance is validated by an average Dice score of 0.763 and a Jaccard index of 0.639, indicating accurate amyloid deposit segmentation, particularly impressive given the limited supervision. Patient-wise cross-validation further confirms its robustness, showing strong generalization to unseen cases and reliable discrimination of relevant structures in complex histological environments.

Taken together, these findings validate the core premise of our pipeline: that accurate segmentation of histological pathology can be achieved through a carefully designed augmentation framework, even when GT annotations are indirect. By leveraging CAMs not only as weak labels but as anchors for object-centric augmentation, we demonstrate a scalable strategy for training robust segmentation models in data-limited medical imaging scenarios.

### 3.3. Morphometric Profiling of Amyloid Plaques via Particle Analysis

The final stage of our analysis delved into the multifaceted morphometric characterization of ThioS-stained amyloid plaques, providing a nuanced perspective on their size, shape complexity, geometric symmetry, and staining intensity. By leveraging an extensive suite of 19 parameters extracted from segmented deposits, we explored structural variations across brain regions and cognitive status, revealing latent morphological patterns through dimensionality reduction and statistical comparison. PCA revealed three principal axes of morphometric variation (PC1–3), each capturing distinct subsets of deposit morphology with biologically interpretable architectural traits.

PC1, which captured the largest share of morphometric variance, predominantly reflected differences in plaque size, suggesting that spatial extent is the most salient morphological feature distinguishing amyloid deposits and potentially influencing their biological impact. Among the morphometric parameters, several size-related features, such as deposit and core area, perimeter, and convex hull-based descriptors, showed consistent co-loading on PC1, supporting a dominant size-related component. Notably, these traits were log-transformed to account for the known log-normal distribution of amyloid plaque sizes in AD, which likely reflects volume-proportional growth patterns [14,105,106]. In line with this trend, integrated density, representing cumulative staining intensity, also aligned with this axis, linking fluorescence accumulation to plaque size, as larger deposits typically accumulate a greater amount of fluorescence signal. Collectively, these parameters capture the overall size and spatial extent of deposits, dimensions that, as revealed in the results, were elevated in the groups with dementia, particularly in the parietal region, suggesting an increased plaque burden associated with dementia. This aligns with previous neuropathological findings linking cortical amyloid load to advanced disease stages and cognitive decline [4].

PC2 was dominated by structural complexity and spatial heterogeneity metrics, shaped by non-linear descriptors such as fractal dimension and lacunarity, alongside shape compactness measures like circularity and solidity. Fractal dimension, a scale-invariant measure quantifying self-similarity and organization, provides insights into structural complexity and is commonly applied to both cellular [107,108,109,110] and non-cellular structures, such as Aβ deposits in AD [11,111], which in this study was positively weighted in PC2. Thus, our findings are partly consistent with previous reports of increased fractal dimension in advanced stages of dementia [11], particularly given the PC2 increase observed in the parietal region. However, the decrease observed in the temporal lobe may point to region-specific differences. It is also important to note that our efforts to exclude vascular structures associated with plaques during segmentation may have influenced this metric, as such structures are likely to affect the apparent structural complexity captured by fractal analysis. In contrast, lacunarity was represented with a strong negative weight in PC2, which increased in the parietal region in the group with dementia, while it decreased in the temporal region. This regional variability may reflect both discontinuities in diffuse, looser structures and differences in subregional patterns within more complex plaque types. In the current analysis, roughness, reflecting deposit boundary irregularity, was negatively weighted in PC2, while circularity and solidity were strongly positively weighted, as their higher values indicate compactness, suggesting denser, morphologically simpler plaques. Notably, circularity has previously been used to classify ThioS-positive plaques by morphology [112]. Given that PC2 increased in the parietal region of dementia cases and decreased in the temporal region, this pattern may indicate a regional shift toward more compact and geometrically regular plaques in the parietal cortex in dementia, whereas plaques in the temporal cortex may retain more irregular, fragmented morphologies. Importantly, the diffuseness index, while technically an intensity-derived metric, quantifying the proportion of weakly stained areas relative to the total deposit area [113], loaded negatively onto this axis, indicating its role in capturing the degree of spatial spread and lack of compactness. PC2 scores were consistently highest in the dementia parietal group and lowest in the dementia temporal group, reflecting regionally divergent shifts in plaque morphology. These shifts involved not only size-related increases but also opposing trends in parameters associated with structural irregularity, compactness, and shape complexity. Such contrasting patterns may point to region-specific pathological trajectories, potentially shaped by differential plaque maturation dynamics, tissue organization, or local microenvironmental influences.

PC3 captured global geometric aspects of plaque morphology, emphasizing spatial symmetry and elongation as key characteristics. Elevated span ratio and max/min radius values reflected more anisotropic, elongated plaque shapes, while low hull circularity values reflected departures from global circular symmetry. Although less commonly assessed in traditional neuropathology (despite prior observations of region-specific patterns in plaque elongation [52]), these features offered additional insight into plaque morphotypes. Notably, PC3 was significantly elevated in both groups with dementia, driven by increases in elongation measures and decreases in symmetry, as it was positively weighted by span ratio and max/min radius and negatively by hull circularity, suggesting a morphological shift toward more elongated and asymmetrical plaques in dementia. This pattern may reflect underlying pathological processes affecting plaque growth dynamics, interactions with the vasculature, or microenvironmental factors that promote anisotropic deposition processes in the diseased brain.

From a biological perspective, the observed morphometric shifts may reflect region-specific tissue architecture, differential plaque maturation stages, or distinct mechanisms of amyloid aggregation and clearance. By applying a joint statistical modeling approach, these multifactorial influences may have been partially disentangled, allowing for the characterization of both global patterns and group-specific morphotypes. Importantly, our analysis underscores the value of incorporating non-traditional descriptors, such as fractal and convex hull-based parameters, which capture subtle structural features beyond conventional size metrics. These advanced measures may provide sensitive readouts for stratifying plaque subtypes or monitoring disease progression.

While the number of analyzed deposits was high, the relatively small number of donor cases (*n* = 7) warrants extreme caution in interpreting group-level trends. All statistical examinations presented in this study must therefore be considered strictly exploratory and overinterpretation at this scale would be fundamentally misleading. The dataset’s narrow scope inherently precludes deeper population-level inference. The main objective of these analyses is not to establish definitive, robust biomarker-level associations, but to illustrate the technical feasibility, practical applicability, and scalability of our segmentation framework. In this light, our morphometric analysis should be regarded primarily as demonstrative, with severely limited interpretability in the context of disease progression. The heterogeneity of cortical pathology is well-documented, and such a small and non-representative donor sample inevitably limits the generalizability and statistical reliability of any observed patterns.

While the consistent region- and dementia-associated patterns across all three PCs are promising and support the utility of high-dimensional morphometric profiling as a complementary tool in neuropathological and imaging-based studies, their statistical robustness remains fundamentally constrained by the dataset size. These findings are intended to motivate, rather than conclude, and larger, more representative cohorts, including multi-region studies, will be essential to validate, refine, and expand these proof-of-concept analyses and explore their true translational potential.

## 4. Materials and Methods

### 4.1. Brain Sections

For the WSSS and subsequent morphometric analysis of amyloid pathology using ThioS staining, 7–8 μm thick paraffin-embedded brain tissue sections were used [114]. The samples from parietal and temporal cortices (or subfields of these areas) of elderly subjects without dementia and with AD were obtained from the Netherlands Brain Bank (Nederlandse Hersenbank/Netherlands Institute for Neuroscience, Meibergdreef 47, 1105 BA Amsterdam, The Netherlands). The samples were collected from donors for or from whom a written informed consent for a brain autopsy had been obtained, and permission was granted for the use of tissue samples and for the anonymous use of clinical information (Project 598/2009). The tissue samples were supplemented with the following clinical/pathological information: autopsy code, age, gender, diagnosis, Braak stage, post-mortem delay, cerebrospinal fluid pH, brain mass, Apolipoprotein E, brain area/region; some of these are included in Supplementary Table S6.

Out of the various brain regions examined, only sections from the temporal and parietal lobes provided sufficient quantity and quality for morphometric analysis. Comparable images could not be obtained during the initial examinations from other available regions (thalamus, subthalamus, locus coeruleus, pons, and hippocampus) and from samples of subjects in Braak 0 and Braak 1 stages, due to the absence of well-identifiable plaques or ThioS-positive parenchymal amyloid structures. For each case, a single section from the superior temporal gyrus (temporal lobe) and one from the superior parietal gyrus (parietal lobe) were analyzed, both from subjects with and without dementia, resulting in a total of fourteen sections from seven individuals (two Braak 2 (no dementia), one Braak 5, and four Braak 6 (with dementia)).

### 4.2. ThioS Staining and Epifluorescence Microscopy for Amyloid Detection

ThioS is a widely used non-specific fluorescent histological dye used to detect various forms of tissue amyloid and pre-amyloid deposits, including amyloid plaques [18]. It binds non-selectively to the characteristic β-sheet conformations of amyloid fibrils and other proteins and emits green fluorescent light [16,17,18]. The sections were deparaffinized, rehydrated, and used in fluorescent microscopic histochemistry. Briefly, the sections (7–8 μm) were first treated three times for 15 min each with cyclohexane isomers of xylene, washed in absolute ethanol for 3 × 2 min, and subjected to a descending ethanol series (1-1 min in 96%, 70%, and 50% alcohol, a total of 3 min), followed by placing the sections in distilled water (minimum 1 min) and stored there until staining. ThioS staining protocol was carried out based on the method of Krutsay [23]. The deparaffinized sections were placed in a 1 g/10 mL solution of ThioS-staining mixture (Reanal, Budapest, Hungary) in distilled water and left at room temperature for 10 min, making sure that the tissue samples were completely immersed. The dissolved staining solution was filtered several times through filter paper before each use. This was followed by a short alcohol differentiation and then a rinse in distilled water. The sections were coverslipped with a water-based cover medium (Vectashield, Vector Laboratories, Peterborough, UK) and examined under a fluorescence microscope. To prevent fading of the samples and photolysis of the dye, the ThioS-solution, and the stained sample slides were protected from light and stored in a cool (4 °C) dark place until the start of the microscopic examination, which was always carried out within two weeks.

Amyloid aggregates, ranging from well-developed senile plaques to smaller, diffuse extracellular deposits were detected using a Leica DMLB epifluorescence microscope (Leica Microsystems CMS GmbH, Wetzlar, Germany). A 40× apochromatic objective lens with a 0.75 numerical aperture and a lens system corrected for an infinite tube length and 0.17 mm-thick cover glass was used (40×/0.75 ∞/0.17). Green-channel, high-resolution digital images of the affected regions were recorded using a Leica DFC7000 T CCD camera and LAS X Leica Application Suite X computer software (version 3.9.28093.0) (Leica Microsystems CMS GmbH, Wetzlar, Germany) with a specified 45 ms exposure time. Efforts were made during the image acquisition process to systematically divide tissue sections on the slide into sub-areas, aiming to minimize image overlaps. Micrographs were saved as TIFF files with a resolution of 72 dpi (1920 × 1440 pixels) and labeled with scale bars.

### 4.3. Preprocessing Fluorescent Micrographs: Uneven Illumination, Background Estimation, and Annotation Preparation

Correcting for non-uniform intensity in ThioS-stained micrographs was essential for reliable quantitative measurements. This illumination gradient introduced variations that could interfere with downstream image processing steps [115]. When fluorescence intensity decreases radially from the optical axis, it can lead to variations in the detected intensity of objects, such as amyloid deposits, across different image regions [21]. Similarly, BG estimation is a critical step in both conventional and automated fluorescent microscopic analysis, essential for detecting the photophysical signals of fluorophore-labeled objects, as BG noise from sources such as out-of-focus fluorescence, detector noise, stray light, and autofluorescence often contributes to the detected signal [116]. Both the illumination gradient and BG noise affect later stages of the workflow, influencing segmentation, morphological analysis, and intensity measurements. The illumination gradient causes regional intensity changes, while BG noise introduces global discrepancies, both affecting downstream analysis. To correct intensity nonuniformity in fluorescence microscopy, flat-field correction (white referencing) is commonly used, removing both illumination gradients and lens contaminants. This method requires a uniformly fluorescent reference image (e.g., an empty field of view or free fluorescent dye), which is used to normalize experimental micrographs [21,115,117]. However, obtaining an accurate reference image is challenging, and it is difficult to precisely reproduce the experimental conditions, even when using the same microscope settings, illumination intensity, and exposure time. This is due to factors such as device anomalies, sample preparation processes, and the behavior of fluorescent stains in the chemical environment of the real samples [115]. As an alternative, retrospective correction methods can estimate illumination bias directly from the acquired images. A simple approach, pseudo-flat-field correction, involves smoothing each image with a large-kernel filter to create a smoothed version, which is then used for normalization. This method assumes uniform signal distribution and does not correct the dust specks [115]. More robust multi-image-based approaches generate an illumination correction function (ICF, vignetting function) by averaging multiple images, followed by median filtering to refine the estimate [21,115,117,118,119]. Our approach, adapted from prior robust methods, applies retrospective multi-image illumination correction to fluorescence images by converting them to 8-bit grayscale and computing the ICF by averaging the intensities of 45 individually selected micrographs containing mostly clear tissue areas from multiple experimental subjects, captured at random positions. The averaged image was then refined using Gaussian blur and median filtering, preserving only larger gradients necessary for correction, then each image was corrected through division by the ICF and pixel scaling with its average value.

Standard BG correction methods based on intensity thresholding [120], are sensitive to changes in the FG intensity distribution. They require a bimodal intensity histogram for reliable BG estimation, but in fluorescence imaging, overlapping FG and BG distributions complicate this approach, especially for amyloid structures with varying intensities and low signal-to-noise ratios. Morphological methods based on structuring elements (e.g., rolling ball method) [121], depend on object size-related parameters and do not estimate BG distributions, making them unsuitable for dealing with the wide size range and locally variable structures of ThioS-positive objects. Instead of relying on a single BG value, the Silver Mountain Operator (SMO) [22] method improves BG representation by directly restoring an unbiased (though not complete) BG distribution from the image. It selects representative BG pixels through a robust statistical procedure, leveraging the absence of local spatial correlation between them. The method involves simple mathematical operations and an averaging kernel, with filter size as its only parameter. In essence, the algorithm computes a moving average of the normalized intensity gradients after optional smoothing and then derives the length of these average vectors [22].

**Figure 6:**
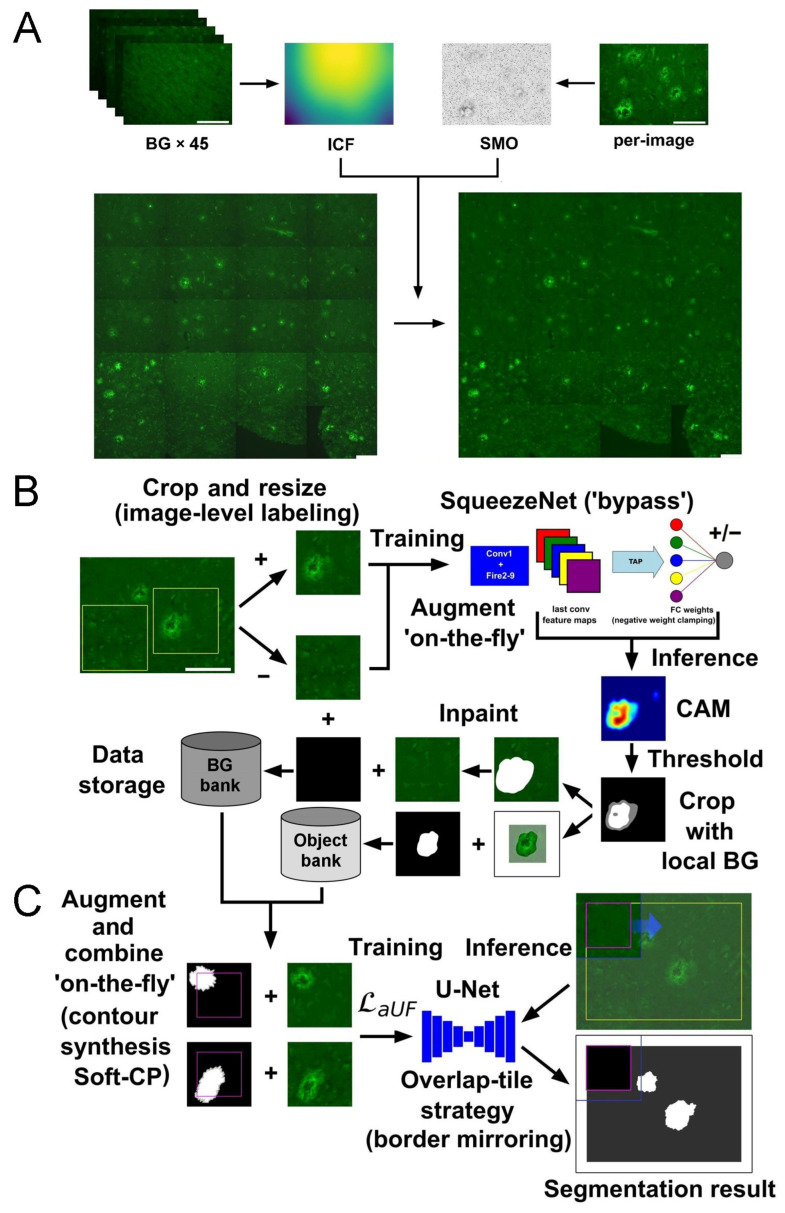
Overview of the three-stage image-processing and training pipeline for weakly supervised amyloid plaque segmentation approach. (**A**) First stage: preprocessing includes illumination correction using an Illumination Correction Function (ICF) and background (BG) estimation via the Silver Mountain Operator (SMO), improving input image quality for downstream tasks. Representative examples show raw (left) vs. corrected (right) image tiles. (**B**) Second stage: image-level labeled data is cropped, resized, and augmented for training a TAP-SqueezeNet binary classifier for the presence or absence of parenchymal amyloid pathology using on-the-fly augmentations. Class activation maps (CAMs) are generated via negative weight clamping, resized, scaled, and thresholded. FG objects are cropped with local BG, and BG images are restored via image inpainting and saved into databanks for downstream use. (**C**) Third stage: segmented object masks and inpainted BGs are augmented and combined using Soft-Copy and Soft-Paste (Soft-CP) and contour synthesis with corresponding trimaps as pseudo-labels. These synthetic examples are used to train a U-Net segmentation model, optimized with an asymmetric unified focal loss (ℒ_aUF_). Final inference employs an overlap-tile strategy with border mirroring to produce full-image segmentation masks. This pipeline enables automated learning of spatial features that distinguish parenchymal pathology from vasculature and BG, while accounting for high morphological variability and weak supervision constraints. Scale bar: 100 μm (applicable to all magnifications, with proportional scaling).

For SMO, a 7-pixel averaging window was used without prior smoothing, and the 5th percentile threshold was chosen. The method for BG extraction was applied after illumination correction, using only the unmasked regions of the corrected images, where saturated pixels (≥255) were excluded, and empty-slide areas (≤5) were also masked. The median of the BG distribution, extracted from the masked images, was subtracted from the entire unmasked image, centering the histogram of the BG regions around zero, while empty-slide regions exhibited extreme negative values, as expected. The images were then rescaled to a 0–255 grayscale range for saving as 8-bit TIFF files for further processing (Figure 6). All correction procedures were implemented using standard Python (version 3.10) libraries such as NumPy, OpenCV [122,123,124] and the SMO Python package version (https://github.com/maurosilber/smo, accessed on 15 February 2025) [22], executed within the Google Colaboratory cloud environment (https://colab.research.google.com, accessed on 15 February 2025) [125].

Following the intensity adjustments, 1607 corrected ThioS-stained fluorescence micrographs (1920 × 1440 resolution) were sampled using ImageJ (version 1.47) [126] where a square region of interest (ROI) of 800 × 800 pixels (chosen based on the typical size range and density of plaques) was manually placed on selected regions, cropped and saved for further analysis with image patch-level binary annotations (1 for the presence of parenchymal pathology, 0 for its absence); vascular elements and residual artifacts were present in both groups and although non-overlapping regions were prioritized, complete non-overlap was impractical due to image size and plaque positioning. The dataset included 2 subjects without dementia and 5 with dementia, each contributing two sections from two distinct brain regions. Positive and negative image patches were systematically extracted from the full-size micrograph images of each subject’s brain sections. Positive patches were selected from regions containing visible ThioS-positive parenchymal amyloid pathology, while negative patches were sampled from areas lacking parenchymal deposits. These negative regions could include BG tissue or vascular amyloid pathology. This patch-level sampling strategy ensured that both classes were represented within each section, allowing for balanced and anatomically diverse training data. The final training dataset, consisting of 2978 positive and 3350 negative image patches for parenchymal pathology (a total of 6329 cropped images) was prepared for deep learning-based image classification tasks. Pixel-wise binary segmentation masks were created on the full images (1920 × 1440) through extensive manual segmentation, where ImageJ tools such as thresholding, morphological operations, and filtering were used to simplify the process, but despite these simplifications, the process still required significant manual effort, including visual inspection, object selection, and adjustments, making it extremely costly, time-consuming and labor-intensive to achieve reliable results; these segmentation masks will be used for the final WSSS evaluation.

### 4.4. Implementation of SqueezeNet-Based CAM for Weakly Supervised Localization

Deep learning aids medical image segmentation using costly dense pixel-level labels; weak supervision pipelines use image-level labels like the CAM method but struggle to delineate lesion boundaries accurately. CAM highlights subregions of an image that contribute most to the prediction by computing a weighted sum of the final convolutional feature maps, with weights derived from the classification layer following a global pooling operation. GAP condenses each feature map into a scalar, forming a pooled vector combined with class-specific weights to generate class scores. The learned class-specific weights are applied directly to the spatially informative feature maps to produce CAMs, which are then upsampled, typically via bilinear interpolation, into heatmaps that highlight areas linked to each class prediction and enable the identification of discriminative image regions with respect to classification outcomes [26]. However, this method provides only coarse-grained localization due to the limited mapping resolution of feature maps, and the global pooling layer imposes architectural constraints by requiring it to directly precede the final classification layer, often necessitating substantial modifications [26,59].

Among CAM-based WSOL methods, we adopted the approach of [59] for its simplicity and effective handling of global pooling issues, specifically using the TAP layer and negative weight clamping (excluding their percentile-based thresholding). TAP averages only activations above a threshold, balancing the broad coverage of GAP with the focused precision of GMP to improve spatial localization. Clamping negative weights to zero during CAM generation suppresses BG signals found in less discriminative object parts without adding training complexity (like erasing methods) or noise [59]. A key limitation of CAM localization is the low spatial (mapping) resolution of final convolutional feature maps. To address this and enable CAM-based localization, ref. [26] removed convolutional, pooling, and fully connected layers in models like AlexNet-GAP, VGGnet-GAP, and GoogLeNet-GAP, increasing mapping resolution (227 × 227 → 13 × 13 and 224 × 224 → 14 × 14 instead of 6 × 6 and 7 × 7), though this may reduce classification performance [58]. SqueezeNet, by delaying downsampling, achieves a similar resolution by default (224 × 224 → 13 × 13; in our case, 572 × 572 → 34 × 34). We applied minor changes replacing GAP with TAP and adding a sigmoid activation for binary classification. To better preserve resolution and align with U-Net-based refinement [75], 800 × 800 input images were resized to 572 × 572 and then normalized to [0, 1]. The SqueezeNet authors proposed three variants (Vanilla, Simple Bypass, and Complex Bypass) and found Simple Bypass most effective by adding skip connections around fire modules (3, 5, 7, 9) to learn residual functions and bypass bottlenecks, boosting accuracy and regularization without increasing model size; we adopted it for our task [56].

The SqueezeNet model, extended with a TAP layer, was trained from scratch on cropped patches derived from histopathological micrographs and corresponding binary labels indicating the presence or absence of parenchymal amyloid deposits. The training was performed using the root mean square propagation (RMSprop) optimizer with a fixed learning rate of 5 × 10^−6^ and a batch size of 64, over 800 epochs, aiming to minimize the binary cross-entropy loss (ℒ_BC_), commonly used in binary classification tasks to measure the difference between the true labels (*y*) and the predicted probabilities (*p*). The formula for the ℒ_BC_: −(1/*n*)∑*^n^_i_*_=1_[*y_i_*log(*p_i_*) + (1 − *y_i_*)log(1 − *p_i_*)]. The best-performing model was retained based on the lowest validation loss across all epochs.

Data augmentation comprised standard geometric transformations (rotation, shearing, zooming, flipping), complemented by mixup regularization, and was applied on-the-fly during training. Mixup coefficients (λ) were sampled from a symmetric Beta distribution, Beta(α = 8.0, β = 8.0), when inputs belonged to the same class, and from Beta(α = 0.2, β = 0.2) when combining samples across classes. Although inter-class interpolation can improve generalization [89], this strategy was suboptimal in our setting due to the presence of pathologically relevant but weakly staining regions. Mixing positive signals with non-pathological samples produced attenuated images that visually resembled naturally weak positives but received disproportionately reduced soft labels, causing ambiguity in supervision (similar effect to manifold intrusion). Model classification performance was assessed using patient-wise 7-fold cross-validation, where each fold corresponded to a distinct subject. This evaluation strategy was chosen to prevent data leakage across subjects and to simulate real-world scenarios where predictions must generalize to unseen individuals. Following the training cycles, average performance metrics across the seven folds were computed and considered representative of overall model performance. For each fold, the following standard classification metrics were calculated based on the confusion matrix elements derived from image-level predictions (TP_cls_: true positives, TN_cls_: true negatives, FP_cls_: false positives, FN_cls_: false negatives):

- Accuracy, defined as TP_cls_/(TP_cls_ + TN_cls_ + FP_cls_ + FN_cls_), measures the overall proportion of correctly classified image patches, regardless of class. It provides a general indication of the model’s reliability in distinguishing between positive and negative instances.
- Precision, defined as TP_cls_/(TP_cls_ + FP_cls_), quantifies the proportion of true positive predictions among all patches predicted as positive, indicating how many of the identified amyloid-positive regions were correctly detected.
- Recall, calculated as TP_cls_/(TP_cls_ + FN_cls_), measures the capacity of the model to correctly identify all truly positive instances; i.e., the proportion of actual amyloid-containing patches that were successfully detected.
- F1-score expresses the trade-off between precision and recall through their harmonic mean, computed as 2TP_cls_/(2TP_cls_ + FP_cls_ + FN_cls_), and proves especially informative in imbalanced classification contexts.

Additionally, during each cross-validation iteration, 10% of the training data, randomly sampled from the combined six training folds, was set aside as a development (or validation) set. This subset served to monitor training progress and to detect signs of overfitting. Model checkpoints were saved throughout the 800 training epochs, and for each iteration, the model state achieving the lowest validation loss on the development set was selected for final evaluation on the corresponding held-out test subject. CAMs were subsequently extracted from the final convolutional layers to enable spatial localization of discriminative features without requiring major architectural modifications (Figure 6). The model and the training pipeline were implemented in Python using TensorFlow, along with additional libraries for data preprocessing, augmentation, and training [122,123,124,127] and the high-level computations were performed on an NVIDIA GeForce RTX 4090 GPU with 24 GB of memory.

### 4.5. Implementation of U-Net Segmentation with Advanced Object-Level Augmentation

For the final image segmentation task, the U-Net was chosen for its effectiveness with limited data. To improve generalization, extensive object-level augmentation strategies were applied during training, as robust augmentation is key for optimal performance [75,81]. CAMs from amyloid-positive images (34 × 34, via SqueezeNet) were upsampled (572 × 572) and normalized to the [0, 1] interval, using per-image maxima [26]; minima defaulted to 0 due to negative weight clamping [59].

Pixel-level pseudo-masks with corresponding image patches were created using a simple two-step thresholding: CAMs > 0.5 defined FG, from which unique objects with their local context were extracted based on contours (cv2.findContours) [124], and saved into an object-level image bank. To synthesize BG-only scenes, CAMs > 0.2 were dilated (25-pixel circular structuring element) to expand buffer zones, removed from the images, and resulting irregular holes inpainted using Shift-Map algorithm (OpenCV 4.2.0) [124], which reconstructs missing regions by finding dominant patch offsets and optimizing a Markov Random Field energy function [128] without training additional deep learning models [98,129]. The completed images were placed into a unified BG bank with the original BG set. This approach maximizes environmental diversity and contextual variation for object insertion, supporting the creation of large-scale synthetic data [96,98,100,101,129,130]. This modular pipeline decouples images into object and BG components, enabling on-the-fly object-level augmentation. During U-Net training, 0–5 randomly selected object instances were inserted into these BG scenes, addressing the class imbalance. Segmentation masks are handled similarly, allowing combinatorial data synthesis, and augmenting the boundaries between objects and BGs for increased diversity and precise control over object-boundary transitions [96,100,101].

To further enhance the diversity of object boundaries, contour perturbation is integrated into the object-level augmentation pipeline for training a U-Net using CAM-derived pseudo-masks. Specifically, contour synthesis is applied to thresholded pseudo-object masks prior to cut-and-paste augmentation. When cutting out these object regions from their local BG patches, the synthesized contours are used both to update the masks and to crop the corresponding grayscale image regions.

Our method builds upon the idea of Restorable Contour Synthesis [131], originally designed to produce synthetic segmentation masks with restorable GT approximations, with its potential for augmentation suggested. The average of multiple such synthetic contours remains a faithful approximation of the original, enabling robust augmentation while maintaining structural plausibility [131]. The perturbation mechanism of the polygonal keypoint approximation is modified for better structural integrity and increased variability. Instead of injecting Gaussian noise scaled to keypoint distances in arbitrary directions [131], displacement is constrained to directions perpendicular to the line connecting two neighbors of the current point, approximating the local contour tangent. This avoids self-intersections and maintains coherent shape outlines even with larger global noise parameters (standard deviation). In practice, key points are sampled with gaps of 5–10 pixels for the polygonal approximation, with the size of the gap directly influencing the magnitude of the noise applied to the points.

To ensure natural integration, the Soft-CP blending strategy [101] was applied during insertion, which smooths object mask edges through erosion and dilation operations, with 5 iterations for each chosen for this work, and weights pixel values based on distance from the contour boundary to ensure a seamless transition. However, the dilated and eroded pixel areas of the mask were ignored during training (similar to a trimap), and, unlike the original Soft-CP method [101], the blending technique was applied only to the input grayscale image without using the soft masks as GT.

The U-Net model and the training process were implemented using standard Python libraries [122,124] and TensorFlow [127]. Training utilized the adaptive moment estimation (Adam) optimizer (learning rate: 0.001, β_1_ = 0.9, β_2_ = 0.999, ε = 1 × 10^−7^) [132], minimizing an asymmetric unified focal loss function (ℒ_aUF_) [133]. This loss function generalizes distribution-based, region-based, and compound losses (e.g., cross-entropy, Dice, and Combo) into a unified framework to address class imbalance, with fewer hyperparameters by grouping functionally equivalent terms, while its asymmetric design promotes FG enhancement and BG suppression [133]. ℒ_aUF_ includes three hyperparameters: λ, which controls the relative weighting between the two constituent loss components; δ, which adjusts the balance between negative and positive examples; and γ is a focusing parameter for simultaneous BG suppression and rare class enhancement. In this study, we set λ = 0.5, δ = 0.6, and γ = 0.5. The asymmetric unified focal loss function is defined as

ℒ_aUF_ = λℒ_maF_ + (1 − λ)ℒ_maFT_ (1)

where ℒ_maF_ is the modified asymmetric focal loss and ℒ_maFT_ is the modified asymmetric focal Tversky loss:

ℒ_maF_ = −(δ/*N*)*y_i:r_*log(*p_t,r_*) − [(1 − δ)/*N*]∑*_c≠r_*(1 − *p_t,c_*)^γ^log(*p_t,r_*) (2)

ℒ_maFT_ = ∑*_c≠r_*(1 − mTI) + ∑*_c=r_*(1 − mTI)^1 − γ^, (3)

where N is the total number of pixels; *y*, and *p* are the GT labels and predicted values; *p_t_* is the probability of predicting the GT class; *i*, *c*, and *r* are the pixel, class and rare class indices; mTI is the modified Tversky index:

mTI = ∑*^N^*_*i*__=1_*p*_0_*ᵢg*_0_*ᵢ*/(∑*^N^*_*i*__=1_*p*_0_*ᵢg*_0_*ᵢ* + δ∑*^N^*_*i*__=1_*p*_0_*ᵢg*_1_*ᵢ* + (1 − δ)∑*^N^*_*i*__=1_*p*_1_*ᵢg*_0_*ᵢ*), (4)

where *p*_1*i*_, *p*_0*i*_ are predicted probabilities for FG/BG; *g*_1*i*_, *g*_0*i*_ are the corresponding GT indicators (1 if true, 0 otherwise) [133]. The loss function was adapted for the binary segmentation task, with modifications made to handle the presence of ignored pixels by excluding them from the calculations. The training was conducted over 30 epochs with a batch size of 16, using augmented pseudo-masks derived from CAM models as GT. During testing and inference, the full-size micrographs were reflected at the borders to avoid empty regions at the edges. The images were then divided into overlapping tiles; each was resized to the input dimensions for prediction. The predictions for each tile were reassembled into a segmentation mask corresponding to the full micrograph. Postprocessing steps including thresholding (0.5), hole filling, and size exclusion (<3500 pixels) to filter out smaller particles were applied, while objects touching or intersecting the image boundaries were excluded during particle extraction (Figure 6).

Rather than exhaustively retraining baseline or ablation variants for the segmentation pipeline, we focused on the per-fold evaluation of the complete framework using independent pixel-level GT annotations of entire images. Given the considerable computational demand of our group k-fold cross-validation setup and the additional challenges posed by dataset-specific factors, such as severe class imbalance, distributional distortions from patch resampling, a large proportion of negative patches in the original patch set, redundancy due to tile overlap, and inconsistencies from semi-automated annotations, all of which complicate fair comparison and baseline construction under conventional dense segmentation settings, we considered this evaluation strategy more appropriate for demonstrating the viability and practical applicability of our weak supervision pipeline. Segmentation performance was evaluated using patient-wise 7-fold cross-validation, where each fold represented a distinct subject. Importantly, the same subject-wise fold partitions established during the classification step were retained to ensure methodological consistency. Specifically, CAMs produced by the TAP-SqueezeNet classifier were used to generate pseudo-labels for the U-Net segmentation model training, and this mapping respected the original fold assignments, ensuring that CAMs derived from a given subject were only used within their respective training folds. This design maintained strict data separation across subjects and allowed a direct correspondence between classification-derived supervision and subsequent segmentation learning. In each cross-validation iteration, the U-Net was trained on six folds and evaluated on the remaining held-out fold. Performance metrics were computed independently for each fold, and final results were reported as the average across all seven folds. Segmentation performance was evaluated using a set of standard pixel-level evaluation metrics derived from the confusion matrix elements computed over segmentation masks (TP_seg_: true positives; TN_seg_: true negatives; FP_seg_: false positives; FN_seg_: false negatives), including both overlap-based and error-based measures:

- Dice coefficient, defined as 2TP_seg_/(2TP_seg_ + FP_seg_ +FN_seg_), quantifies the spatial overlap between predicted and GT segmentation masks. It reflects both precision and recall and is widely used in biomedical image segmentation to assess agreement.
- Jaccard index (also known as Intersection over Union), given by TP_seg_/(TP_seg_ + FP_seg_ + FN_seg_), provides a stricter measure of overlap than the Dice coefficient. It evaluates the proportion of shared positive predictions relative to the union of predicted and actual positives.
- Recall, calculated as TP_seg_/(TP_seg_ + FN_seg_), measures the ability of the model to correctly detect all amyloid-positive pixels.
- Pixel-level accuracy (PA), computed as (TP_seg_ + TN_seg_)/(TP_seg_ + TN_seg_ + FP_seg_ + FN_seg_), assesses the overall correctness of predictions across all pixels, including both FG and BG. However, in cases of strong class imbalance, where BG pixels vastly outnumber FG pixels, accuracy may be inflated and fail to reflect true segmentation performance.
- Specificity, calculated as TN_seg_/(TN_seg_ + FP_seg_), reflects the model’s ability to correctly identify BG (non-deposit) pixels, reducing the likelihood of over-segmentation.
- Precision, defined as TP_seg_/(TP_seg_ + FP_seg_), indicates the fraction of true deposit pixels among all those classified as deposits.
- Negative Predictive Value (NPV), calculated as TN_seg_/(TN_seg_ + FN_seg_), indicates the proportion of correctly classified BG pixels among all BG predictions.
- False Positive Rate (FPR), given by FP_seg_/(FP_seg_ + TN_seg_), quantifies the proportion of BG pixels incorrectly labeled as deposits.
- False Discovery Rate (FDR), calculated as FP_seg_/(FP_seg_ + TP_seg_), indicates the proportion of incorrect deposit predictions among all positive predictions.
- False Negative Rate (FNR), defined as FN_seg_/(FN_seg_ + TP_seg_), represents the fraction of actual deposit pixels that were missed by the model.
- False Omission Rate (FOR), defined as FN_seg_/(FN_seg_ + TN_seg_), measures the proportion of missed FG pixels among all predicted BG pixels.

In each cross-validation round, a randomly selected 10% subset of the training folds was used as a development set to monitor training dynamics. This set underwent the same data augmentation pipeline as the training data to ensure the presence of synthetic boundary variations, rather than relying on raw thresholded CAMs. The development set was used exclusively to track stable training trends; model checkpointing was not based on its performance, and the model saved at the 30th epoch was consistently used for evaluation. We implemented the model and training procedures in Python with TensorFlow, and other libraries for preprocessing, augmentation, and training [122,123,124,127]. High-level computations were carried out on an NVIDIA GeForce RTX 4090 GPU with 24 GB of memory.

### 4.6. Implementation of Morphometric Profiling of Amyloid Plaques via Particle Analysis

Morphometric analysis of parenchymal amyloid deposits was performed in subjects with and without dementia, focusing on parietal and temporal cortices. Compact and diffuse subregions were delineated using intensity thresholds at the 80th and 50th percentiles of pixel intensities within the predicted mask (from the final model) of each collected deposit, following prior work for calculating the diffuseness index using a single histological stain [113]. For each deposit, two binarized images based on these thresholds were generated and used alongside corresponding grayscale regions for object-specific morphometric analysis. In total, 19 parameters were measured using the computer programs ImageJ and FracLac for ImageJ (https://imagej.nih.gov/ij/docs/guide/user-guide.pdf and https://imagej.net/ij/plugins/fraclac/FLHelp/Introduction.htm; accessed on 15 February 2025) [107,108,126]. While 7 of these parameters have been previously examined in the context of amyloid pathology [11,14,105,106,112,113], the remaining 12 have primarily been applied in general structural–morphometric studies of biological tissues, offering novel perspectives on the microstructural organization of amyloid plaque. The extracted morphometric parameters were categorized into four major groups: (1) log-transformed, size-related metrics, including deposit area, compact core area, perimeter, and convex hull metrics such as hull area, hull perimeter, bounding circle diameter, maximum span, and mean radius; (2) structural complexity descriptors, including fractal dimension, lacunarity, roughness, circularity, and solidity; (3) geometric and symmetry features, including span ratio, max/min radii ratio, and hull circularity; (4) intensity-based characteristics, including mean gray value, integrated density, and diffuseness index. These categories collectively provide insights into the geometry, structural complexity, morphological variability, and staining characteristics of amyloid deposits.

To capture size-related variation, several deposit features were assessed, which were log-transformed (base 10) to achieve more symmetric distributions for PCA, account for the log-normal size distribution of Aβ-plaques in AD, consistent with a stochastic, volume-proportional growth model around a log-scale mean, likely driven by a porous, sponge-like structure in equilibrium with its surroundings. This cross-sectional pattern, supported by histology and 3D reconstructions, reflects the true volumetric properties of near-isotropic objects [14,105,106]. Deposit and compact area, determined by two predefined threshold values, were calculated from FG pixel counts in binarized images, and then converted to square micrometers, while the perimeter was computed as the length of the composite contour outlining the deposit area (in micrometers). Integrated density, defined as deposit area × mean gray value, was also log-transformed for PCA, as it reflects size-dependent total intensity within the deposit area. The size-related parameters were further expanded to include convex hull-based measurements. The convex hull refers to the smallest convex polygon that encloses the deposit area and several parameters were calculated based on this shape: hull area in square micrometers, and hull perimeter, diameter of the bounding circle (the smallest circle enclosing the hull), maximum span (the greatest distance between two points on the convex hull), and mean radius (the average distance from the center of mass of the hull to an outer point), all measured in micrometers.

To further understand the spatial structure of plaques, we analyzed size-invariant, internal architecture-related morphometric descriptors. Fractal dimension and lacunarity were computed to quantify the spatial complexity and heterogeneity of the binarized amyloid deposits. Fractal dimension was estimated using a box-counting approach, where the slope of a log–log regression between the number of FG pixels within boxes and the scanning box size was calculated. Lacunarity assessed the distribution of gaps and discontinuities within the deposit structure, with higher values indicating greater heterogeneity and lower values suggesting uniformity. Both measures were obtained using the FracLac plugin for ImageJ [107,108], applying the binary scanning method on thresholded images, using a series of grids with varying sizes. The “White Scan Background” option was fixed, and the grid calibers were determined by applying a power series for the box size scale. Measurements were performed on 12 randomized grid placements, and the results were obtained by averaging these measurements [107,108]. In addition to these metrics, the following parameters, though derived from measurements related to plaque size, focus on assessing size-invariant features that capture structural complexity and irregularity, namely: roughness (the ratio of perimeter to hull perimeter) reflects border jaggedness; circularity (4π × [deposit area]/[perimeter]^2^) quantifies similarity to a perfect circle; solidity (the ratio of deposit area to hull area) indicates compactness; and the diffuseness index (calculated as [deposit area − compact area]/deposit area, following [113]) represents the proportion of weakly stained regions.

Furthermore, global geometric and overall shape characteristics of deposits were quantified using convex hull-based parameters that capture aspects of spatial symmetry and elongation. These included span ratio (the ratio of the major to minor axis of the convex hull), max/min radius (distance ratios from the center of mass to boundary points), and hull circularity (calculated as 4π × hull area/hull perimeter^2^), collectively providing a comprehensive representation of plaque shape variability.

Subsequently, a comparative statistical analysis was performed based on deposit-level morphometric parameters across the two cortical lobes (parietal and temporal) and the presence or absence of dementia. This yielded four distinct experimental groups: (1) parietal + non-dementia, (2) parietal + dementia, (3) temporal + non-dementia, and (4) temporal + dementia. Prior to dimensionality reduction, all parameters were standardized using z-score transformation (StandardScaler) [134]. When the measured parameters contained zero values (compact area), a constant value of 1 was uniformly added across all observations to enable subsequent log-transformation [135,136]. PCA [134] was then performed to reduce the dimensionality of the dataset and to identify dominant patterns in the morphometric data. The PCs accounting for the highest proportion of explained variance were selected for further group-wise comparisons. The average values of the main PCs were compared across the four groups using ANOVA, and significant differences were further investigated with a Tukey post hoc test to compare pairs of groups. All statistical analyses were carried out using Python (libraries: scikit-learn, pandas, scipy, matplotlib) [122,134,137,138,139] and R (stats) [140], ensuring consistency and reproducibility.

## 5. Conclusions

In this study, we introduced a weakly supervised image analysis pipeline that enables accurate, robust, and interpretable segmentation of ThioS-positive Aβ plaques in human AD brain tissue without requiring labor-intensive pixel-level annotations. Our approach uniquely integrates robust preprocessing, the generation of CAM-derived spatial priors from a lightweight SqueezeNet classifier trained solely on patch-wise image labels, and a U-Net-based segmentation refinement step using synthetic, pseudo-labeled data with extensive object-level augmentation, without relying on manual delineations.

Importantly, the resulting models generalized well to unseen subjects’ original whole-image data under patient-wise group k-fold cross-validation, demonstrating strong alignment with manual GT. They successfully identified biologically meaningful, disease-relevant patterns by separating parenchymal amyloid pathology from vascular structures and staining artifacts, a distinction that ThioS staining protocols alone cannot reliably achieve due to high background noise and non-specific binding properties. Despite a limited donor pool, intrinsic boundary ambiguities, marked structural heterogeneity in deposit morphology, and variable staining patterns, our pipeline consistently captured plaque-specific semantic features while effectively excluding irrelevant tissue components.

Beyond significantly reducing annotation burden, our weakly supervised segmentation framework enables automated, object-level identification and detailed morphometric profiling of individual amyloid plaques, effectively distinguishing them from other pathological elements, confounding objects, and background staining. This approach circumvents the need for manual selection and overcomes the inherent limitations of the staining procedure itself. Our pipeline thus facilitates large-scale, precise histopathological assessment in AD, as demonstrated by morphometric analyses revealing region- and cognitive status-associated variations in plaque size, structural complexity, and geometry. The ability to extract such biologically meaningful per-object features from specimens demonstrates the practical utility of our method and its potential to contribute to mechanistic insights into AD pathology; however, these morphometric findings should be interpreted with caution due to the limited number of donor cases.

All experiments in this study were conducted exclusively on ThioS-stained cortical tissue micrographs. However, the modular design of our pipeline, including intensity normalization, preprocessing, model architectures, CAM-based localization, and patch-wise training, is not inherently stain-specific. With suitable adjustments, the method can be adapted to other contexts; nevertheless, different target applications inherently involve unique challenges and trade-offs, requiring tailored adaptations of the framework to effectively address their specific objectives and limitations. This framework can be extended to broader donor cohort and brain region coverage, additional fluorescent staining protocols, different histopathological targets and potentially alternative imaging modalities, including 3D datasets, such as Congo red staining, transgenic animal models, in vivo approaches, or Lewy body pathology in Parkinson’s disease, etc., where detailed manual annotations remain scarce.

Altogether, our results demonstrate that carefully designed weak supervision can yield segmentation models that are not only computationally efficient, but also capable of uncovering clinically relevant and biologically interpretable morphological signatures. This positions our pipeline as a scalable, annotation-efficient, and adaptable tool for digital neuropathology in data-constrained clinical settings.

## Data Availability

The data are contained in the article and its Supplementary Files.

## Supplementary Materials

The following supporting information can be downloaded at https://www.mdpi.com/article/10.3390/ijms26157134/s1.

## Abbreviations

AD	Alzheimer’s disease
Adam	adaptive moment estimation
ANOVA	analysis of variance
Aβ	amyloid-β
BG	background
CAM	class activation maps/mapping
CNNs	convolutional neural networks
FDR	false discovery rate
FG	foreground
FN_cls/seg_	false negatives for classification/segmentation tasks
FNR	false negative rate
FOR	false omission rate
FP_cls/seg_	false positives for classification/segmentation tasks
FPR	false positive rate
GAP	global average pooling
GMP	global max pooling
GT	ground truth
ICF	illumination correction function
mTI	modified Tversky index
NPV	negative predictive value
PA	pixel-level accuracy
PCA	principal component analysis
PCs	principal components
RMSprop	root mean square propagation
ROI	region of interest
SMO	Silver Mountain Operator
Soft-CP	soft-copy and soft-paste
TAP	thresholded average pooling
ThioS	thioflavin-S
TN_cls/seg_	true negatives for classification/segmentation tasks
TP_cls/seg_	true positives for classification/segmentation tasks
WSOL	weakly supervised object localization
WSSS	weakly supervised semantic segmentation
ℒ_aUF_	asymmetric unified focal loss
ℒ_BC_	binary cross-entropy loss
ℒ_maF_	modified asymmetric focal loss
ℒ_maFT_	modified asymmetric focal Tversky loss
