# Supplementary material for: Automated Segmentation and Morphometric Analysis of Thioflavin-S-Stained Amyloid Deposits in Alzheimer’s Disease Brains and Age-Matched Controls Using Weakly Supervised Deep Learning
Source: Int J Mol Sci. 2025 Jul 24;26(15):7134. doi: 10.3390/ijms26157134 (PMC12346360; doi:10.3390/ijms26157134)
Supplement: Supplementary file 1 [file ijms-26-07134-s001.zip › Supplementary Tables (S1–S6).docx]

**Supplementary Tables (S1–S6)**

**Supplementary Table S1. Evaluation of SqueezeNet across 7-fold patient-wise cross-validation**

| **Metrics** | **Loss (ℒ_BC_)** | **Accuracy** | **Precision** | **Recall** | **F1-score** | **TP_cls_** | **FP_cls_** | **TN_cls_** | **FN_cls_** |
| --- | --- | --- | --- | --- | --- | --- | --- | --- | --- |
| **S1** | 0.064 | 0.979 | 0.993 | 0.954 | 0.973 | 270 | 2 | 430 | 13 |
| **S2** | 0.051 | 0.976 | 0.982 | 0.965 | 0.973 | 277 | 5 | 330 | 10 |
| **S3** | 0.053 | 0.985 | 0.978 | 0.994 | 0.986 | 702 | 16 | 593 | 4 |
| **S4** | 0.075 | 0.981 | 0.963 | 0.986 | 0.974 | 494 | 19 | 832 | 7 |
| **S5** | 0.052 | 0.977 | 1.000 | 0.957 | 0.978 | 336 | 0 | 311 | 15 |
| **S6** | 0.058 | 0.979 | 0.985 | 0.962 | 0.973 | 329 | 5 | 504 | 13 |
| **S7** | 0.066 | 0.979 | 0.996 | 0.971 | 0.983 | 494 | 2 | 301 | 15 |
| **Average**  **±**  **SD** | 0.060 ± 0.009 | 0.979  ±  0.003 | 0.985  ±  0.013 | 0.970  ±  0.015 | 0.977 ± 0.005 |  |  |  |  |

Results are reported for each test fold, corresponding to one held-out subject per iteration. Subject-wise test-folds: S1: S05/044; S2: S05/174; S3: S00/085; S4: S06/282; S5: S00/141; S6: S05/265; S7: S02/234. BC: Binary Crossentropy, TP_cls_: True Positives, FP_cls_: False Positives, TN_cls_: True Negatives, FN_cls_: False Negatives; ℒ_BC_: binary cross-entropy loss.

**Supplementary Table S2. Evaluation of U-Net across 7-fold patient-wise cross-validation**

| **Metrics** | **Dice** | **Jaccard index** | **Recall** | **Accura-cy (PA)** | **Specif-icity** | **Precision** | **NPV** | **FPR** | **FDR** | **FNR** | **FOR** |
| --- | --- | --- | --- | --- | --- | --- | --- | --- | --- | --- | --- |
| **S1** | 0.79 | 0.668 | 0.695 | 0.993 | 0.999 | 0.949 | 0.993 | 0.001 | 0.051 | 0.305 | 0.007 |
| **S2** | 0.823 | 0.712 | 0.861 | 0.994 | 0.997 | 0.815 | 0.997 | 0.003 | 0.185 | 0.139 | 0.003 |
| **S3** | 0.723 | 0.586 | 0.607 | 0.987 | 0.999 | 0.955 | 0.988 | 0.001 | 0.045 | 0.393 | 0.012 |
| **S4** | 0.83 | 0.724 | 0.774 | 0.994 | 0.999 | 0.926 | 0.995 | 0.001 | 0.074 | 0.226 | 0.005 |
| **S5** | 0.662 | 0.537 | 0.571 | 0.989 | 0.998 | 0.926 | 0.99 | 0.002 | 0.074 | 0.429 | 0.01 |
| **S6** | 0.729 | 0.59 | 0.81 | 0.99 | 0.993 | 0.699 | 0.996 | 0.007 | 0.301 | 0.19 | 0.004 |
| **S7** | 0.782 | 0.654 | 0.73 | 0.984 | 0.995 | 0.872 | 0.988 | 0.005 | 0.128 | 0.27 | 0.012 |
| **Average**  **±**  **SD** | 0.763 ± 0.061 | 0.639  ±  0.07 | 0.721 ± 0.105 | 0.990  ±  0.004 | 0.997  ±  0.002 | 0.877  ±  0.093 | 0.992 ± 0.004 | 0.003 ± 0.002 | 0.123 ± 0.093 | 0.279 ± 0.105 | 0.008 ± 0.004 |

Results are reported for each test fold, corresponding to one held-out subject per iteration. Subject-wise test-folds: S1: S05/044; S2: S05/174; S3: S00/085; S4: S06/282; S5: S00/141; S6: S05/265; S7: S02/234. Jaccard index (also known as Intersect over Union), PA: Pixel-level Accuracy, NPV: Negative Predictive Value, FPR: False Positive Rate, FDR: False Discovery Rate, FNR: False Negative Rate, FOR: False Omission Rate.

**Supplementary Table S3. PCA component matrix showing loadings for morphometric features (coefficients of linear combination)**

|  | **PC1** | **PC2** | **PC3** |
| --- | --- | --- | --- |
| **Area** | 0.315331* | 0.03825 | 0.015109 |
| **Integrated Density** | 0.313937* | 0.066851 | 0.03992 |
| **Mean Gray Value** | 0.128959 | 0.287417* | 0.249961* |
| **Perimeter** | 0.28724* | -0.18604 | -0.06546 |
| **Compact Area** | 0.274224* | 0.176746 | 0.115567 |
| **Diffuseness Index** | -0.15823 | -0.31045* | -0.23279 |
| **Fractal Dimension** | 0.201069 | 0.313247* | -0.02082 |
| **Lacunarity** | -0.07369 | -0.36984* | -0.02449 |
| **Circularity** | -0.0854 | 0.372379* | 0.15918 |
| **Roughness** | 0.234115* | -0.20448* | -0.17582 |
| **Density (Solidity)** | 0.113979 | 0.394863* | 0.017796 |
| **Span Ratio (major/minor axis)** | -0.0751 | -0.10599 | 0.519861* |
| **Maximum Span Across Hull** | 0.28806* | -0.16582 | 0.142867 |
| **Hull Area** | 0.303791* | -0.12744 | 0.020387 |
| **Hull Perimeter** | 0.298698* | -0.14516 | 0.072276 |
| **Hull Circularity** | 0.153809 | 0.154413 | -0.51143* |
| **Max/Min Radii** | -0.13409 | -0.16272 | 0.46942* |
| **Mean Radius** | 0.294311* | -0.1553 | 0.092996 |
| **Diameter of Bounding Circle** | 0.288618* | -0.16568 | 0.13928 |

Dominant parameters of the principal components (PCs) are reported. * indicate the highest absolute loading values for each variable across the principal components, highlighting the most influential features contributing to the respective PC.

**Supplementary Table S4. One-way analysis of variance (ANOVA) for principal components (PC1, PC2, and PC3) with group comparisons**

| **PC1** | **Df** | **Sum Sq** | **Mean Sq** | **F value** | **Pr(>F)** |
| --- | --- | --- | --- | --- | --- |
| Groups | 3 | 220 | 73.29 | 7.497 | 0.0000529*** |
| Residuals | 4431 | 43318 | 9.78 |  |  |
| **PC2** | **Df** | **Sum Sq** | **Mean Sq** | **F value** | **Pr(>F)** |
| Groups | 3 | 461 | 153.74 | 33.72 | <2e-16*** |
| Residuals | 4431 | 20200 | 4.56 |  |  |
| **PC3** | **Df** | **Sum Sq** | **Mean Sq** | **F value** | **Pr(>F)** |
| Groups | 3 | 210 | 70.13 | 35.82 | <2e-16*** |
| Residuals | 4431 | 8674 | 1.96 |  |  |

Group comparisons along PC1–PC3 based on ANOVA. Df: degrees of freedom; Sum Sq: sum of squares; Mean Sq: mean square. The F values indicate the test statistics, and Pr(>F) values (i.e., *p*-values) reflect the probability of observing the results under the null hypothesis for each PC. Residuals represent the portion of the total variability in the data that cannot be explained by the group differences. ****p*<0.001.

**Supplementary Table S5. Multiple comparisons of means - Tukey contrasts for group differences along principal components (PC1, PC2, and PC3)**

| **PC1** | **Estimate** | **± SE** | ***t*-value** | **Pr(>\|*t*\|)** |
| --- | --- | --- | --- | --- |
| parietal + non-dementia vs. parietal + dementia | -0.5975 | 0.149 | -4.011 | <0.001*** |
| temporal + dementia vs. parietal + dementia | 0.1 | 0.1079 | 0.927 | 0.784 |
| temporal + non-dementia vs. parietal + dementia | -0.2016 | 0.1633 | -1.235 | 0.595 |
| temporal + dementia vs. parietal + non-dementia | 0.6975 | 0.1535 | 4.543 | <0.001*** |
| temporal + non-dementia vs. parietal + non-dementia | 0.3958 | 0.1965 | 2.015 | 0.176 |
| temporal + non-dementia vs. temporal + dementia | -0.3017 | 0.1675 | -1.801 | 0.264 |
| **PC2** | **Estimate** | **± SE** | ***t*-value** | **Pr(>\|*t*\|)** |
| parietal + non-dementia vs. parietal + dementia | -0.26522 | 0.10172 | -2.607 | 0.0427* |
| temporal + dementia vs. parietal + dementia | -0.73951 | 0.07367 | -10.038 | <0.001*** |
| temporal + non-dementia vs. parietal + dementia | -0.30142 | 0.1115 | -2.703 | 0.0328* |
| temporal + dementia vs. parietal + non-dementia | -0.47429 | 0.10485 | -4.524 | <0.001*** |
| temporal + non-dementia vs. parietal + non-dementia | -0.0362 | 0.13416 | -0.27 | 0.9929 |
| temporal + non-dementia vs. temporal + dementia | 0.43809 | 0.11436 | 3.831 | <0.001*** |
| **PC3** | **Estimate** | **± SE** | ***t*-value** | **Pr(>\|*t*\|)** |
| parietal + non-dementia vs. parietal + dementia | -0.50633 | 0.06666 | -7.596 | <0.001*** |
| temporal + dementia vs. parietal + dementia | -0.25229 | 0.04828 | -5.226 | <0.001*** |
| temporal + non-dementia vs. parietal + dementia | -0.61192 | 0.07306 | -8.375 | <0.001*** |
| temporal + dementia vs. parietal + non-dementia | -0.25404 | 0.06871 | 3.697 | 0.00117** |
| temporal + non-dementia vs. parietal + non-dementia | -0.10559 | 0.08791 | -1.201 | 0.61708 |
| temporal + non-dementia vs. temporal + dementia | -0.35963 | 0.07494 | -4.799 | <0.001*** |

Group comparisons along PC1–PC3 based on Tukey post hoc tests; Estimates ± SE represent the mean differences and their standard errors; the corresponding t-values indicate the test statistics, and Pr(>|*t*|) (i.e., *p-*values) reflect the probability of observing the results under the null hypothesis. **p* < 0.05, ***p* < 0.01, ****p* < 0.001.

**Supplementary Table S6. Clinical characteristics of human subjects included in this study**

| **Subjects (case ID)** | **Age (year)** | **Post-mortem delay (min)** | **Sex** | **Braak stage** |
| --- | --- | --- | --- | --- |
| **Aged persons (non-dementia)** | 81.4 ± 8 | 380 ± 48 |  |  |
| S00/320 | 82 | 330 | f | 1 |
| S05/174 | 86 | 385 | f | 2 |
| S06/282 | 89 | 385 | f | 2 |
| S06/117 | 66 | 465 | m | 0 |
| S07/030 | 84 | 335 | m | 1 |
| **AD persons (dementia)** | 80.2 ± 9 | 325 ± 72 |  |  |
| S00/085 | 80 | 320 | f | 6 |
| S02/234 | 86 | 250 | f | 5 |
| S05/044 | 91 | 345 | f | 6 |
| S05/265 | 64 | 450 | f | 6 |
| S00/141 | 80 | 260 | m | 6 |

Tissue samples were obtained from the Netherlands Brain Bank, Netherlands Institute for Neuroscience, Amsterdam. Averaged data are expressed as mean ± S.D. m: male, f: female.
